# Unraveling Common Patterns and Differences among Cruzipains through Molecular Dynamics Simulations and Structural Analyses

**DOI:** 10.1021/acsomega.5c01876

**Published:** 2025-05-02

**Authors:** Lucianna Helene S. Santos, Augusto César Broilo Campos, Viviane Corrêa Santos, Alexandre Victor Fassio, Maurício G. S. Costa, Rafaela Salgado Ferreira

**Affiliations:** † Institut Pasteur de Montevideo, Mataojo 2020, Montevideo 11400, Uruguay; ‡ Departamento de Bioquímica e Imunologia, Universidade Federal de Minas Gerais, Avenida Antônio Carlos 6627, Belo Horizonte 31270-901, Minas Gerais, Brazil; § Instituto de Física de São Carlos, Universidade de São Paulo, São Carlos, São Paulo 13563-120, Brazil; ∥ Programa de Computação Científica, Vice Presidência de Educação Informação e Comunicação, Fundação Oswaldo Cruz, Rio de Janeiro 21040-900, Brazil

## Abstract

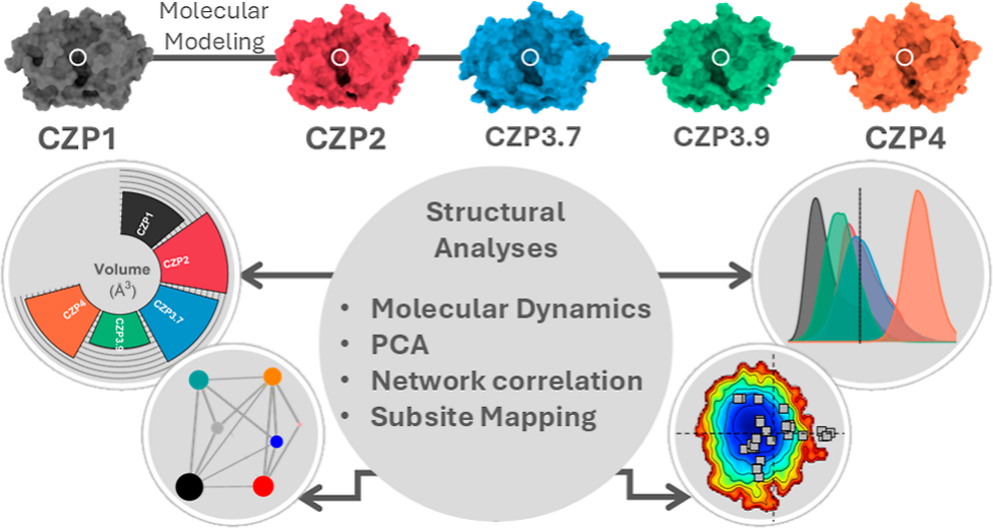

Chagas disease (CD) is a neglected tropical disease for which novel and improved treatments are needed. The cysteine protease Cruzipain is one of the main targets for the development of novel drugs for the treatment of CD. Recent bioinformatics analyses have revealed four Cruzipain subtypes whose active sites differ in key positions for ligand recognition. These analyses suggest a possible effect on the substrate specificity and affinity for ligands. To better investigate the impact of substitutions in Cruzipain subtypes, we employed molecular dynamics simulations and varied structural analyses on representatives of each Cruzipain subtype. Our results indicated that the substitutions did not significantly affect the overall flexibility and conformation of these proteases. In contrast, we observed differences in their active site characteristics, including different electrostatic potentials, cavity volumes, and patterns of interactions with the virtual probes. The distinct alterations in the active site subsites, especially in the S2 subsite, suggest unique functional changes that could affect substrate binding, ligand recognition, and possibly enzymatic effectiveness in various biological situations.

## 1 Introduction

Chagas disease (CD), caused by the protozoan parasite *Trypanosoma cruzi* (*T. cruzi*), is endemic in 21 Latin American countries.^1^ Owing to increased population mobility, CD is currently an emerging health problem in nonendemic countries scattered across North America, Europe, Asia, and Oceania, and it has been detected in 44 countries.^1−5^ It is estimated that at least 6–7 million people worldwide are infected with the parasite.^1,6,7^ The therapeutic arsenal for CD treatment is limited to the nitroheterocyclic drugs benznidazole and nifurtimox, which exhibit several limitations, such as inadequate efficacy during the chronic phase of CD, severe adverse toxic effects, and the presence of resistant strains of *T. cruzi*.^8,9^ Developing new treatments for Chagas disease has been challenging, with a very low throughput of drug candidates, even when compared to other neglected tropical diseases.^10,11^

Cruzipains constitute a multigenic family of cathepsin L-like cysteine proteases from *T. cruzi*. Traditionally, the term “Cruzipain” refers to the native enzyme,^12,13^ and the term cruzain is employed for the recombinant form, which is truncated at the C-terminal domain but preserves proteolytic function.^14^ Cruzipains are expressed in all life *stages of theT. cruzi* cycle and play an important role in metacyclogenesis, host cell invasion, and modulation of the host cell response.^15−21^ Cruzain has been one of the main targets for the development of novel drugs for the treatment of CD.^11,22−25^ Strategies based on designing peptidomimetics, virtual screening, experimental screening, and traditional medicinal chemistry efforts have led to the development of several classes of cruzain inhibitors.^26−34^ Among these inhibitors, there are compounds that are efficacious against *T. cruzi* in vitro and in animal models of infection,^35−39^ and the vinyl sulfone K11777 has progressed to drug candidacy.^40^

Despite decades of effort to develop cruzain inhibitors and understand the biological role of Cruzipains, only recently did we obtain a complete picture of their genomic organization.^41^ Comparison of all Cruzipain sequences from three *T. cruzi* strains (CL Brener, YC6, and Dm28) revealed two Cruzipain families and four Cruzipain subtypes (CZP1–4), whose active sites differed in key positions for ligand recognition. These analyses suggest a possible impact on the substrate specificity and affinity for ligands. In addition, based on transcriptomic analysis in CL Brener, the expression of Cruzipain subtypes varies throughout the *T. cruzi* life cycle.^41^ Similarly, previous qualitative studies with the Dm28c strain detected Family I Cruzipain mRNA only in epimastigotes but found mRNA for Family II Cruzipains in trypomastigotes and amastigotes.^42^ This stage-specific expression pattern may help explain the varying trypanocidal activities observed for the two cruzain-targeting compounds. Compound 8, a competitive cruzain (CZP1) inhibitor with a *K*_i_ of 4.6 μM, showed greater potency against epimastigotes, which express Family I more abundantly. In contrast, its analogue compound 22, a weaker cruzain inhibitor (*K*_i_ = 27 μM), was more effective against trypomastigote and amastigote forms, where Family II Cruzipains are more expressed. Notably, both compounds share the same isoquinoline scaffold yet display distinct activity profiles across parasite stages.^33^ Thus, a better understanding of this protease family is essential to guiding successful medicinal chemistry efforts. To date, the information available on these proteases is very limited. Among the sequences from Family II, only Cruzipain 2, described in 1994,^42^ has been recombinantly expressed and biochemically characterized. Compared to cruzain, CZP2 is less sensitive to E-64, to the mammalian inhibitor cystatin C, and to heparan sulfate.^43,44^ CZP2 also has different substrate specificities, particularly at the S2 subsite.^43,45^ Together with several substitutions detected among the Cruzipain subtypes, these results indicate the relevance of additional experimental and computational studies to better understand this protease family.

Here, we employed molecular dynamics (MD) simulations and varied structural analyses of representatives of each Cruzipain subtype to gain insight into the impact of the substitutions on the flexibility, conformational ensemble, and active site characteristics of these proteases.

## 2 Results and Discussion

### 2.1 Cruzipains Share Conformational Similarity and Rigidity in the Catalytic Domain

**Figure 1:**
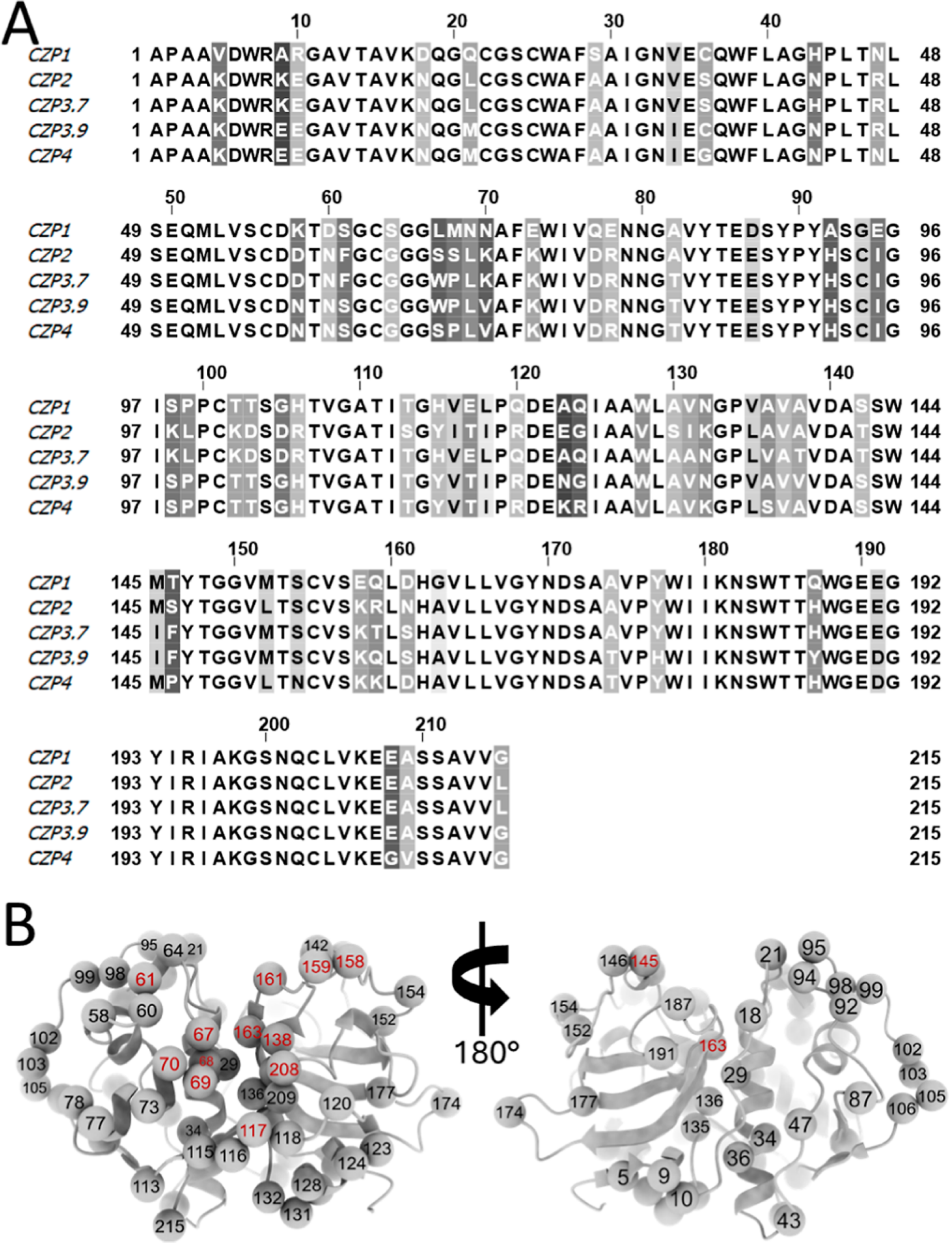
Alignment of the selected Cruzipain sequences and mapping of the substitutions in the structure. (A) Alignment of the catalytic domain of Cruzipain subtypes 1 (CZP1), 2 (CZP2), 3.7 (CZP3.7), 3.9 (CZP3.9), and 4 (CZP4). Amino acid residues that differ from those in any of the other sequences are highlighted in gray. The darker gray coloring indicates a higher sequence variability in the position. (B) Mapping of the substitutions in the catalytic domain of Cruzipains using the CZP1 structure (PDB code 1ME3) as a reference. The alpha-carbons of the amino acid residues substituted in at least one subtype are represented by spheres; the position of the residue is denoted by the number; red numbers indicate residues located at the active site.

We selected five Cruzipain representatives, which were compared throughout this study. These include one member of subtypes 1 (CZP1), 2 (CZP2), and 4 (CZP4) and two members of subtype 3 (CZP3.7 and CZP3.9). Although CZP3.7 and 3.9 were similar enough to be classified within the same subtype, they exhibited differences in the active site, such as in positions 61 and 159, which could significantly impact substrate and ligand recognition, thus justifying the inclusion of both sequences in our analysis. Only the catalytic domain of these sequences was considered since this is the region with higher variability, and it is sufficient for protease activity. Taking CZP1 as a reference, 64 positions were found to vary in at least one of the other four sequences (Figure 1A), of which 13 positions (at residues 61, 67, 68, 69, 70, 117, 138, 145, 158, 159, 161, 163, and 208) were concentrated in the active site, and the remaining positions were spread throughout the whole structure (Figure 1B).

**Figure 2:**
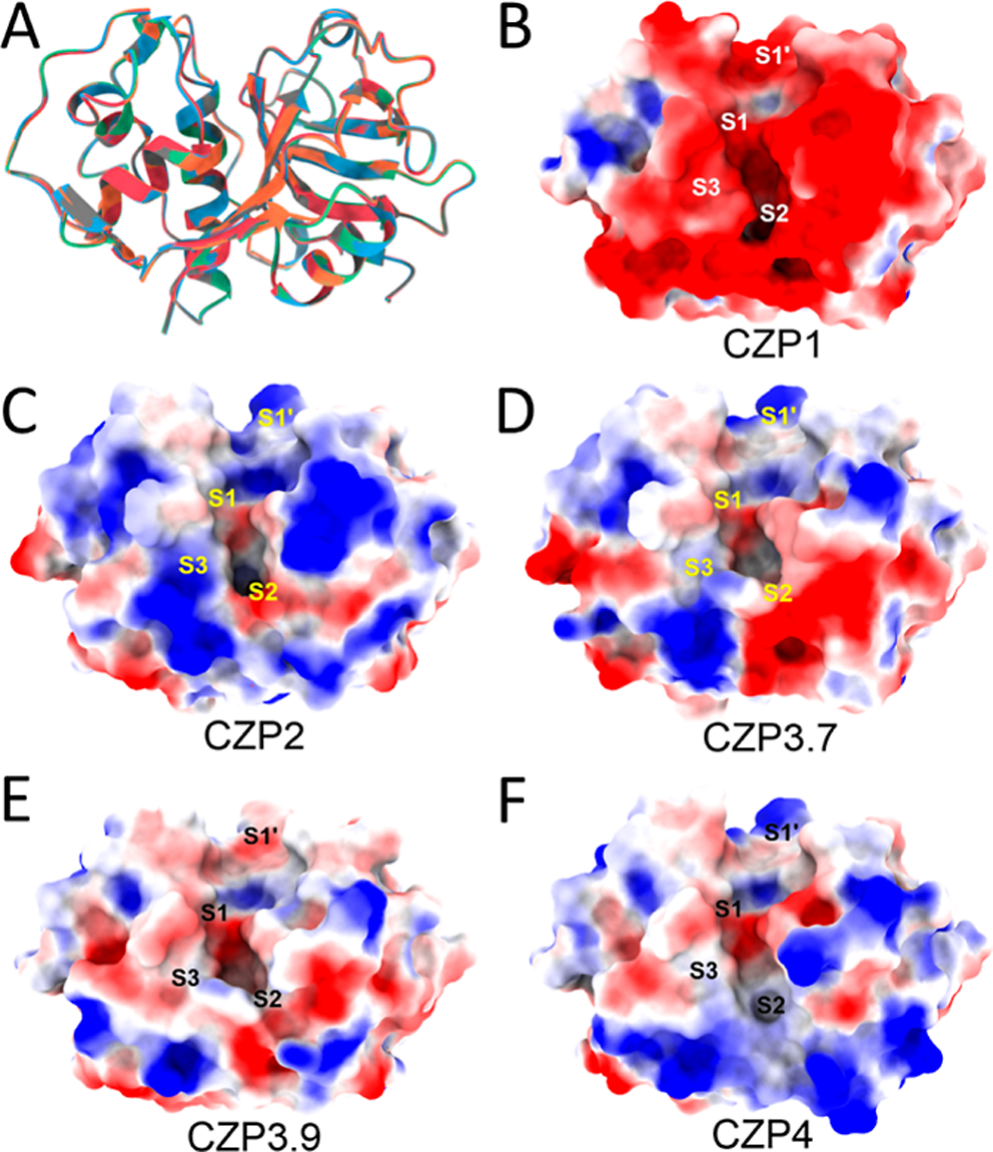
Comparison of the Cruzipain subtypes. (A) The CZP1, CZP2, CZP3.7, CZP3.9, and CZP4 structures superimposed based on their alpha-carbons. RMSD between all the structures was approximately 0.2 Å. Electrostatic surface potentials of CZP1 (B), CZP2 (C), CZP3.7 (D), CZP3.9 (E), and CZP4 (F). The potentials are colored red and blue for negative and positive charges, respectively, and white represents neutral residues.

To further comprehend the effects of these amino acid differences in the catalytic domain, we performed a structural analysis beginning from a cruzain structure obtained by X-ray crystallography (PDB code 1ME3) for CZP1 and from models previously created for the remaining sequences by comparative modeling.^41^ Despite the substitutions being spread over the entire structure, the superimposition of all structures revealed a relatively modest variation, with a root-mean-square deviation (RMSD) of approximately 0.2 Å (Figure 2A). However, the 13 substitutions in the active site region resulted in noticeable variations in both shape and charge among the subtypes (Figure 2B). Electrostatic potential calculations revealed that the CZP1 active site is significantly more negative than the other subtypes. Although the other subtypes did not exhibit a fully positive or negative electrostatic potential, areas that were more positive or neutral around the S2, S3, and S1′ subsites were detected (Figure 2C–F).

Notably, the S2 subsite, known for its significance in ligand selectivity and recognition,^41,46^ exhibited the most noticeable shape variations. The residues Leu67 and Met68, which play a crucial role in defining the subsite in CZP1, are replaced by two serines, Ser67 and Ser68, in CZP2. Therefore, the subsite in CZP2 is enlarged and exhibits a polarity different from that of CZP1 (Figure 2C). In contrast, the presence of Trp67 and Pro68 in CZP3.7 and CZP3.9 leads to a decrease in S2 volume and partial occlusion of the S3 subsite in these subtypes (Figure 2D,E). In CZP4, the substitutions of Ser67, Pro68, and Gly208 lead to a substantial enlargement of S2 (Figure 2F). The Glu208Gly substitution is particularly significant for CZP4 as Glu208 plays a crucial role in binding various ligands.^41,46,47^ When the compound moieties bound in S2 can form a hydrogen bond or an ionic interaction, the Glu208 side chain is oriented toward the binding site. When S2 is occupied by a hydrophobic moiety, Glu208 undergoes a conformational alteration to engage with the solvent.^46^ Thus, replacing a larger residue with a smaller one could significantly affect CZP4’s ability to bind ligands in this specific subsite. Curiously, there was no noticeable impact on the polarity detected in the S1 subsite across the different subtypes. This could be attributed to the presence of catalytic dyad residues inside it. Therefore, this subsite should be under a higher evolutionary pressure than the other subsites; otherwise, the catalytic function could be lost or impaired.

**Figure 3:**
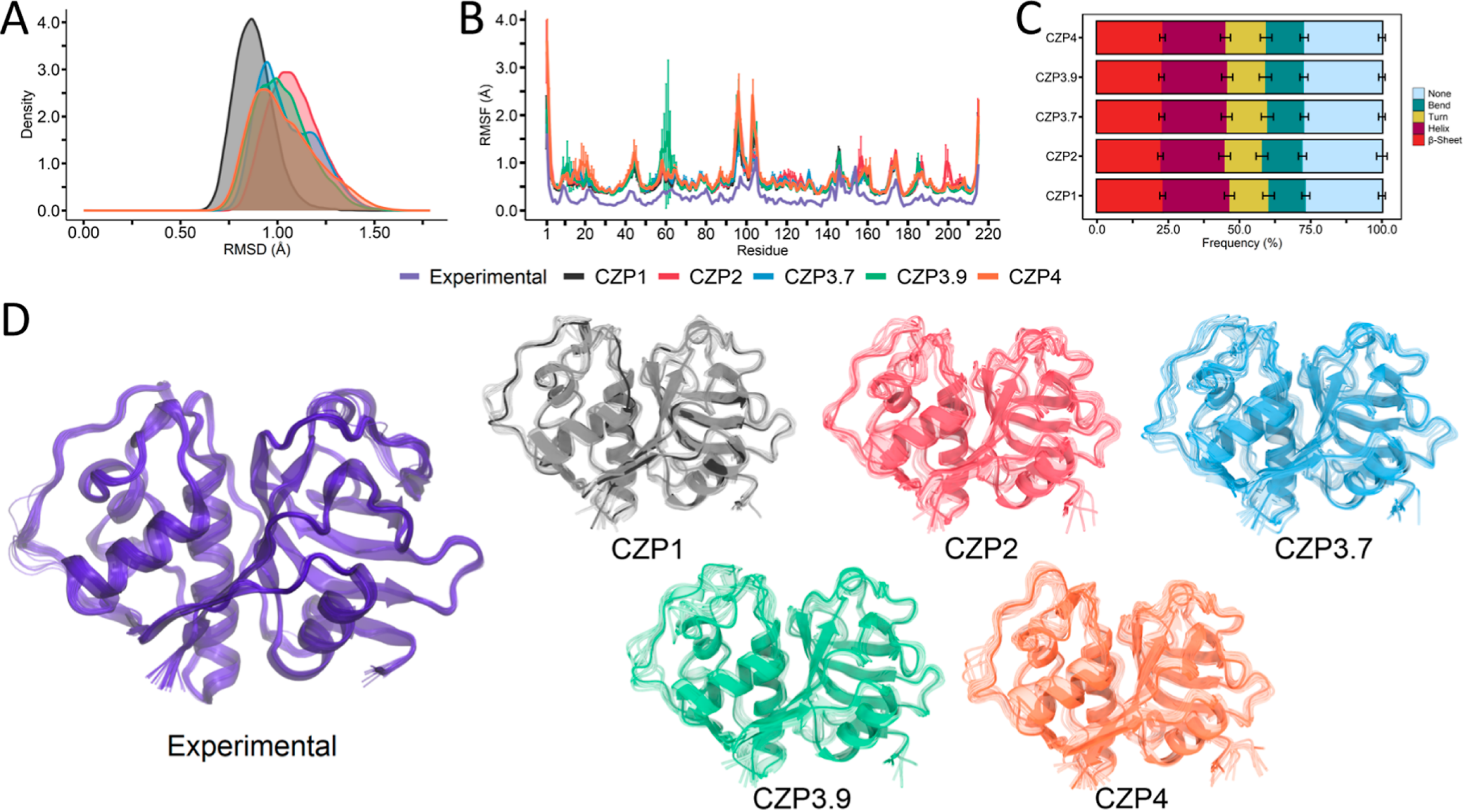
Comparison of molecular descriptors for Cruzipain subtypes. (A) Normalized RMSD distribution for all three replicas for CZP1 (black), CZP2 (red), CZP3.7 (blue), CZP3.9 (green), and CZP4 (orange). (B) RMSFs for all systems and the experimental ensemble from 31 cruzain PDB structures (purple). RMSFs were calculated considering the fluctuations of carbon alpha atoms along the three trajectory replicas. . (C) Average secondary structure content calculated over the three replicates for each system. (D) Representative structures derived from cluster analysis of the trajectories and the experimental ensemble. All systems, except for CZP4, generated 10 representative structures. CZP4, on the other hand, generated 20 structures. The representative structure from the most populous cluster is displayed in a solid color, whereas the remaining representative structures are rendered as transparent.

**Figure 4:**
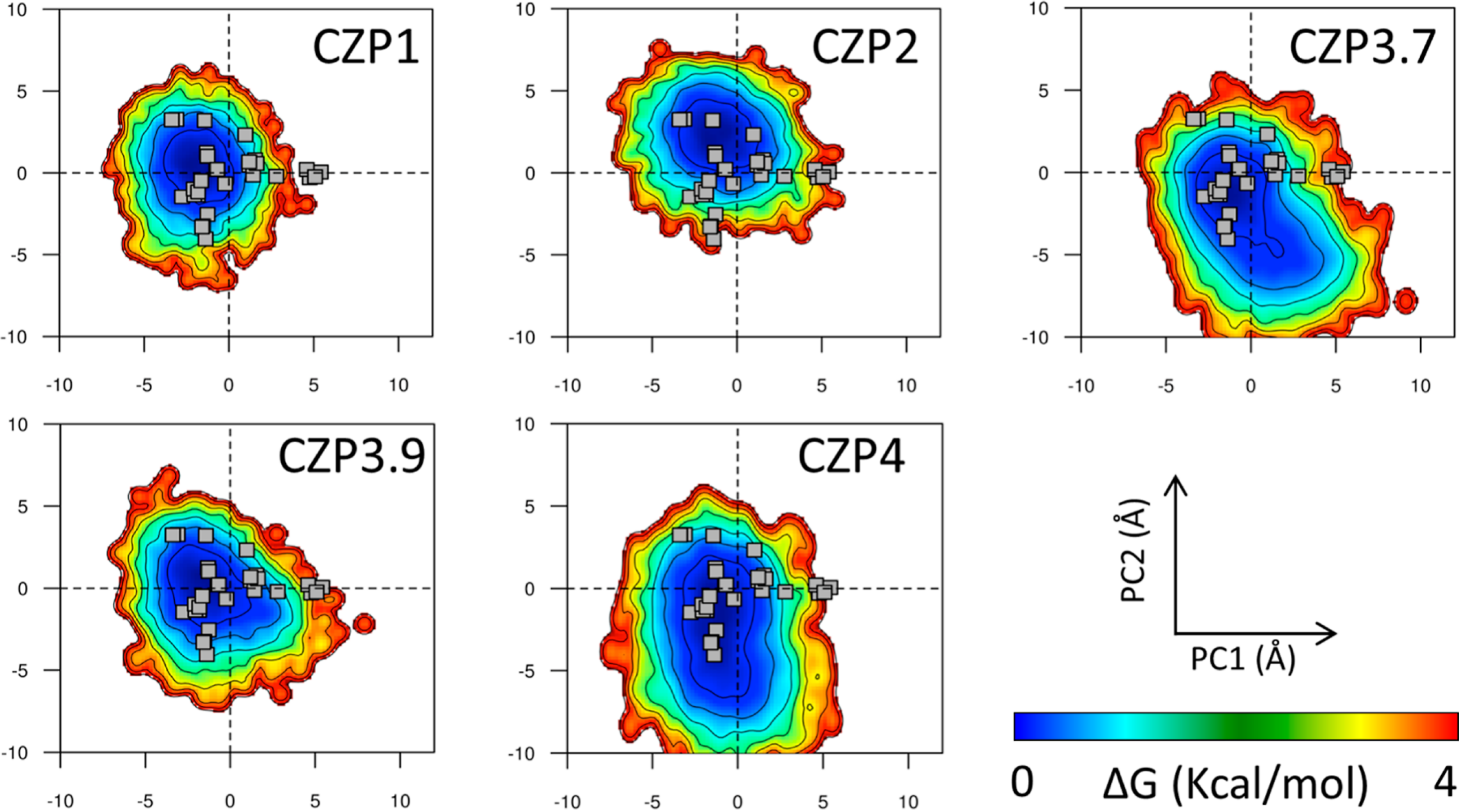
Free energy landscape (FEL) analysis. For each system, the three independent MD replicas were concatenated into a single trajectory and subsequently projected onto the principal components calculated from the CZP experimental ensemble. The projections of CZP experimental structures are shown as gray squares.

Motivated by the observation of different structural features across CZP subtypes, we investigated the conformational variability in the experimental structures and compared it with a set of MD simulations. Three independent 300 ns MD simulations were performed, accounting for a total of 900 ns of simulation time per system. Notably, all subtypes exhibited stability with consistently low deviations (Figure S1), as indicated by the RMSD values ranging from 0.6 to 1.5 Å (Figure 3A). The RMSF profiles were similar for all subtypes, with higher flexibility in the protein loops and terminal regions, as observed in the experimental ensemble (Figure 3B). Indeed, the correlation coefficients between the RMSF profiles for each subtype compared to the experimental ensemble were above 0.7, indicating that MD simulations reproduced the CZP1 flexibility (Table S1). The only exception was CZP3.9, which still exhibited high correlations (0.65), yet lower than other systems, probably because of the peak of fluctuations around residues 60–65. Furthermore, all systems exhibited similar secondary structure compositions (Figure 3C). Consequently, no major disruptions of the overall CZP structure or unfolding were identified because of amino acid substitutions (Figure 3D). This observation reinforces previous studies that demonstrate low flexibility for CZP1^33,48−51^ and the minimal variability observed in the experimental ensemble obtained for this work (Figure 3D). Finally, we calculated the most statistically relevant structural variations found in the experimental ensemble using principal component analysis (PCA). The first two principal components (PC1 and PC2), which describe the structural variations with the largest amplitude, are related to a subtle opening of the active site cavity and loop motions. The projections of the MD snapshots onto these components reveal that all subspaces spanned by the experimental structures were explored during the simulations (Figure 4). CZP4 exhibited the largest range of exploration, which is in line with the largest number of clusters obtained for this subtype. Thus, despite the substitutions found in CZP subtypes, the overall behavior was preserved. Taken together, the results presented in this section reveal that MD simulations for all CZP subtypes explored the conformational variability experimentally found for CZP1.

### 2.2 Network Analysis Indicates Similar Paths and Communities among Cruzipains

**Figure 5:**
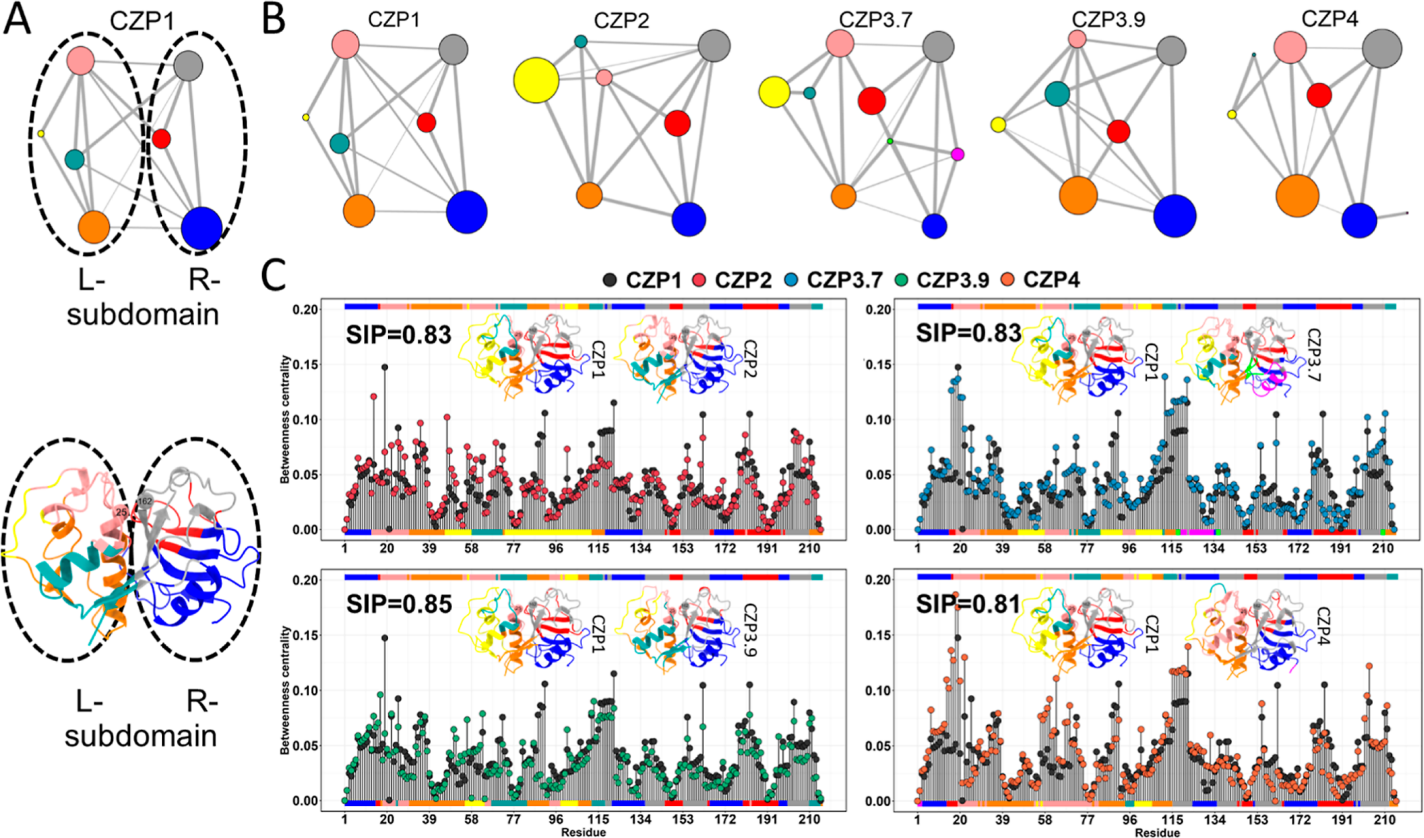
Correlation networks from the Cruzipain subtype simulations. Each community is depicted as a node, with the size of the node proportional to the number of residues it contains. Community connections are shown by edges between nodes. (A) Description of the separation between the L- and R-subdomains using CZP1 as an example. (B) For CZP1, CZP2, and CZP3.9 simulations, the correlation network was clustered in 7 communities, which encompass the whole catalytic domain. For CZP3.7 and CZP4, the correlation network was clustered in 9 and 8 communities, respectively. Communities and respective regions in the subtype structures are indicated by node colors for all simulations. (C) Comparison between the betweenness centrality per residue in CZP1 and other Cruzipains. Node colors are annotated at the margin of the plot, the upper margin for CZP1 and the lower margin for other Cruzipain systems.

As the overall structural and dynamic behaviors were similar for all systems, we proceeded to examine in detail the residue couplings in response to different substitutions by comparing the dynamical cross-correlation matrices (DCCMs). The DCCMs of each system exhibited a notable degree of similarity in terms of correlated motions, with greater intensities observed in the CZP3.9 simulations (Figure S2). Furthermore, in all systems, two well-defined blocks of positively correlated residues were observed, where the first comprised residues 1 to 118 and the second comprised residues 120 to 200. Therefore, the active site region is split into two subdomains, marked by the presence of the catalytic dyad (Cys25 and His162) (Figure 5A). This behavior is also seen in other simulations of cruzain.^52^ Prior studies have classified these dynamic subdomains as the L-subdomain, which includes Cys25, and the R-subdomain, which includes His162.^53,54^

Next, we performed a correlation network analysis to gain further insights into the dynamic couplings obtained in the DCCMs. This analysis enables partitioning of the residues into communities, which are subnetworks of each original network. Nodes in a community have stronger connections with their neighbors than with nodes in other communities. Therefore, this analysis is well suited for the identification of dynamic subdomains. The networks derived from the simulations indicated consistent partitions within the Cruzipains’ structure representing different subtypes, with only a few variations (Figure 5B). Furthermore, we observed that, in general, the R-subdomain contains highly similar communities, except for CZP3.7, while the L-subdomain shows variations in community sizes.

When compared to the other sequences, the R-subdomain of CZP3.7 was split into two additional communities, specifically residues 123–132 (magenta) forming an α helix and residues 135–139 (green), which consists of a short loop near the S2 subsite. Only one substitution, Ala131Val, is exclusively present in CZP3.7 at this node. The green nodes consist of four residues from a β sheet and exhibit a substitution found exclusively in CZP3.7, Val136. In the other subtypes, Ala136 (CZP1, CZP2, and CZP3.9) or Ser136 (CZP4) were present.

As previously mentioned, the community compositions and pattern of connectivity in the L-subdomains exhibited higher variability because of the increased structural heterogeneity observed among the subtypes. Within the L-subdomain, residues 98 to 105 form a loop that stands out. In the CZP1, CZP3.9, and CZP4 subtypes, these residues are mostly clustered together in a single community, represented by the yellow node (Figure 5B). This loop has an identical sequence (SPPCTTSG) and more flexibility in these subtypes (Figure 3B). In contrast, the CZP2 and CZP3.7 subtypes, which contain the sequence KLPCKDSD in this loop, exhibit rigidity in this region, resulting in a bigger community encompassing this loop (Figure 5B). Finally, the CZP4 network distinguishes itself from the others by the substantial size of the pink and orange nodes, which encompass a significant portion of the L-subdomain. The pink node contains a complete α helix, spanning from position 68 to 78, which was subdivided differently in the other subtypes. Therefore, the CZP4 subdomain exhibits more extensive areas of correlated motion than the other subtypes in this region of the L-subdomain.

To examine the importance of each individual residue in the network, the betweenness centrality was computed for each residue (Figure 5C). This metric considers strongly interconnected residues as crucial for the dynamics of residue coupling and communication within the structures. In the CZP1 subtype, residues Gln19, Tyr91, Glu122, His162, and Ser183 exhibited the highest betweenness values (Figure S3A). In the CZP2 subtype, in addition to His162, the highly connected residues identified were Thr14 and Arg47 (Figure S3B). In the CZP3.7 subtype, Val16, Lys17, Asn18, Gln19, Gly20, Leu21, Ile112, Thr113, Gly114, His115, Val116, Glu117, Leu118, Pro119, Gln120, Asp121, Glu122, Cys203, and Ser211 were highly connected residues (Figure S3C). In the CZP3.9 subtype, Lys17, Thr113, and Glu122 exhibited the highest betweenness values (Figure S3D). In the CZP4 subtype, Thr14, Ala15, Val16, Lys17, Asn18, Gln19, Gly20, Cys22, Cys63, Tyr115, Val116, Thr117, Ile118, Pro119, Arg120, Asp121, Glu122, and Cys203 were highly connected residues (Figure S3E).

The presence of residues at the interface of the L- and R-subdomains, including Thr14, Asp18 (Asp18Asn), Gln19, Lys17, Ile112, Thr113, Gly114, His115 (His115Tyr), Val116, Glu117 (Glu117Thr), Leu118 (Leu118Ile), Pro119, His162, Ser183, and Ser211, emphasizes the significance of interdomain signal transmission. Conserved residues located at positions 14, 15, 16, 17, 19, 20, 22, 63, 91, 112, 113, 114, 116, 119, 121, 122, 162, 183, 203, and 211 were more frequently observed as high centrality residues than nonconserved residues at positions 18, 21, 47, 115, 117, 118, and 120, demonstrating that these residue substitutions affect communication pathways in the subtypes. In subtypes CZP3.7 and CZP4, as previously observed with community distribution, numerous residues exhibited elevated centrality values, some located in the subdomain interface region, suggesting the presence of multiple communication pathways in these subtypes encompassing a larger number of residues and diverse regions of the proteins.

Although the residues exhibiting high-density network connections were distinct among the subtypes, overall, the distribution of residues inside the communities and the pattern of connections between them were highly consistent across all systems (Figure 5C). Thus, we utilized the square inner product (SIP) to assess the overall similarity between the betweenness centrality computed for CZP1 and those for other Cruzipains. According to this analysis, high SIP values may be associated with the preservation of communication between residues, even in the presence of substitutions. The SIP values between CZP1 and all the other subtypes were roughly 0.80, suggesting that residue substitutions had an effect, although a modest one, on the overall communication across residues in the catalytic domain for the subtypes when compared to the CZP1 profile. The maximum SIP value of 0.87 occurred between CZP3.7 and CZP4, and the minimum SIP value, 0.75, was observed between CZP2 and CZP4 (Table S2). This aligns well with the previously discussed similarities and disparities in clustering and the community size.

### 2.3 Active Site Analyses Reveal Different Cavity Volumes

Although the MD simulations and network analysis showed that there was a high level of structural similarity among all Cruzipain subtypes, the significant heterogeneity within the active site prompted us to perform more detailed investigations in this region. As previously stated, the substitutions in the active site affect both the electrostatic surface and the formation of its subsite cavities, particularly S2 and S3 (Figure 2). Therefore, we utilized MD trajectories to determine the volume and accessibility of the active site cavity in all subtypes.

**Figure 6:**
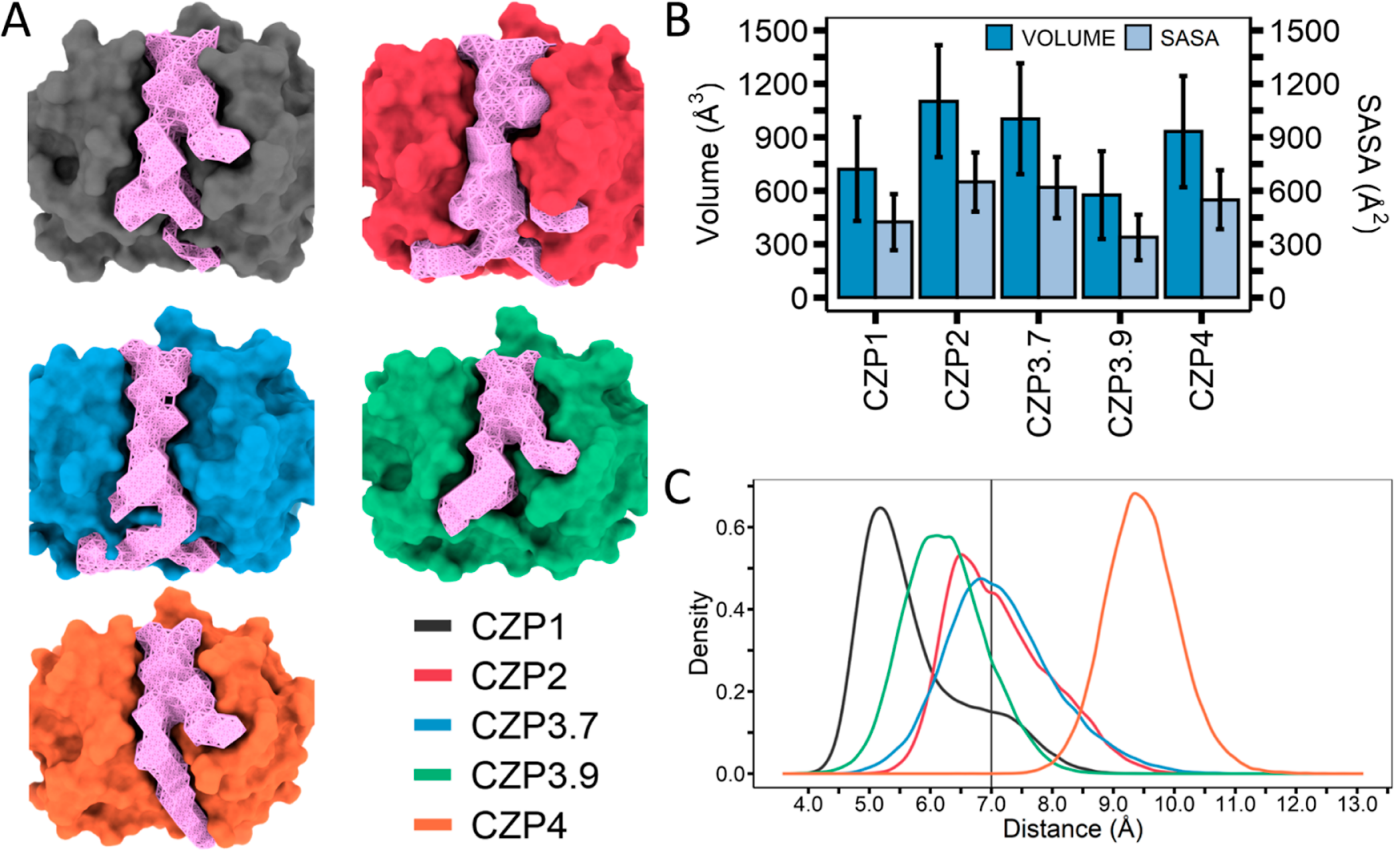
Investigation of the active site cavity of the different Cruzipain subtypes. (A) The active site zone (pink surface) mapped for CZP1 (gray), CZP2 (red), CZP3.7 (blue), CZP3.9 (green), and CZP4 (orange) during the MD simulations. (B) Volume (blue) and SASA (light blue) for each mapped cavity. (C) The inter-residue distance between residues located at positions 67 and 208 measured during the MD simulations. The S2 “gate” is deemed to be open when the distance exceeds 7 Å (black vertical line). The curves were assigned colors based on their respective subtypes.

The active site pocket was characterized using the MDpocket program.^55^ MDpocket automatically chooses pockets from the simulation frames that occur with a frequency of 50% as the default setting. Based on these settings, only the region over the S2 subsite was consistently identified in all Cruzipain subsites, coherently with the generally flat shape of the active site with only the S2 subsite having a distinct shape. Consequently, we reduced the frequency to 10% of the frames to capture all the active sites (Figure 6A).

Notably, CZP2, CZP3.7, and CZP4’s active sites exhibited the largest volumes (about 900 to 1300 Å^3^) and had similar solvent accessible surface areas (SASA) (about 500 to 700 Å^2^) (Figure 6B). These values could be attributed to the enlargement of the pocket resulting from the activation of a “gate” mechanism involving the residues in the S2, leading to a pocket located beyond the S2 subsite. The mechanism previously described by Durrant et al.^56^ elucidates how the residues Leu67 and Glu208, which are near each other, function as a gate in the CZP1 subtype. This gate effectively closes off the region of S2, preventing the formation of an additional cavity. Durrant et al. utilized the distance between residues 67 and 208 as a metric to assess the functioning of this gate during the simulations and determined that the gate is considered open when the distance between residues exceeds 7.0 Å. Using this distance approach, we consistently found that CZP4 retains an open S2 entry throughout the whole simulation. This can be attributed to the replacement of Glu208 with Gly208 (Figure 6C). Notably, CZP2 and CZP3.7 have open gates for around half of the simulation time. For CZP2, a serine residue at position 67 provides a side chain smaller than that of Leu67, primarily leaving S2 in an open conformation. Similarly, in CZP3.7, the distances showed that the gate remained mostly open, even though a tryptophan residue with a bulky side chain is found at position 67. A possible reason for the opening of CZP3.7’s S2 is the presence of Phe61 in the S3 region, leading to the constant relocation of Trp67 to interact with Phe61 (Figure S4). Notably, CZP1 and CZP3.9 have closed gates for most of the simulation time.

### 2.4 Virtual Probes Reveal Variations in Druggability and Interactions at the Subsites of Cruzipain Subtypes

The differences in volume, depth, and polarity observed for the subtypes active sites suggest a possible impact on ligand recognition and druggability. Thus, we used the web server FTMap to probe the protein surface and identify the most druggable subsites of the representative structures of the most populous clusters from MD for each Cruzipain. FTMap results were then analyzed with DrugPy,^57^ a PyMOL plugin, to classify the subsites according to their druggability.

The S2, S1, and S1′ subsites were classified as hotspot areas by FTMap (Table 1 and Figure S5). The probes employed by FTMap were anchored in consensus in these regions, resulting in clusters that contained more than 30 sampled poses each. According to DrugPy, the S1 subsite was druggable in all subtypes, which is coherent with the protease’s active site. S1′ was also deemed to be druggable in all subtypes except for subtype CZP2. Subsites S1 and S1′ were alternately perceived as having low druggability (druggable only by peptides, macrocycles, or charged compounds). The S2 subsite was only druggable in CZP2 and CZP4 and a hotspot in CZP3.7, which is consistent with the presence of the S2 “gate” discussed previously.

**Table 1:** Assessment of the Druggability of Cruzipains’ Cavities by FTMap and DrugPy

	classification^a^		
subtype	hotspots^b^	hotspots and druggable^c^	hotspots and druggable small^d^
CZP1	Thr14 cavity, Asn47/Arg47 cavity	S1, S1′	S1, S1′, Gln37 cavity
CZP2	S1′, Thr14 cavity, Asn47/Arg47 cavity, Arg106 cavity	S2, S1	S1, Gln37 cavity
CZP3.7	S2, Asn47/Arg47 cavity	S1, S1′	S1, S1′, Gln37 cavity
CZP3.9	S1, S1′, Asn47/Arg47 cavity	S1, S1′	S1′, Gln37 cavity
CZP4	Thr14 cavity, Asn47/Arg47 cavity	S2, S1, S1′, Gln37 cavity	S1, Gln37 cavity, Arg106 cavity

a Some cavities were identified with multiple classifications due to the analysis of multiple conformations and different classifications depending on the conformation.

b Cavities classified as hotspots by FTMap but not classified as druggable by DrugPy.

c Cavities classified as druggable by DrugPy and as hotspots by FTMap.

d Druggable small stands for cavities druggable only by peptides, macrocycles, or charged compounds.

Two cavities outside the active site were consistently identified in the L-subdomain. The Gln37 cavity is close to Gly215, the C-terminal residue, and was identified as a hotspot in all of the subtypes. In CZP4, this cavity was also classified as druggable by DrugPy. The Asn47 (or Arg47, depending on the subtype) cavity has been reported as a putative allosteric site in CZP1 before^52^ and was herein identified as a hotspot in all subtypes. The cavity comprises Lys17, Asp18, Asn47, Glu86, and Tyr91 in CZP1; Lys17, Asn18, Asn47, Glu86, and Tyr91 in CZP4; and Lys17, Asn18, Arg47, Glu86, and Tyr91 in CZP2, CZP3.7, and CZP3.9. Lys17 is notably one of the most interconnected residues alongside Asn18 in CZP3.7, whereas this pair also appears in CZP4, with Asn18 being the most prominent in this subtype. In addition, Lys17 is the most connected residue in CZP3.9. In subtypes CZP1 and CZP2, the communications with Lys17 or Asp18 (or Asn18 in CZP2) are less perceived and altered with residues Tyr91 and Arg47, respectively. The presence of these highly connected residues in this region strongly suggests the existence of an allosteric site for all subtypes. Nonetheless, just a singular representative structure of CZP2 and CZP3.7 forms this cavity, rendering it a transitory cavity throughout the simulations, while the SPPCTTSG loop in CZP1, CZP3.9, and CZP4 confers more flexibility in these subtypes as described before and might be the explanation for the constant presence of the Asn47/Arg47 cavity in these structures. The Asn47/Arg47 cavity was classified as a hotspot by FTMap but not classified as druggable by DrugPy for all subtypes. Another two cavities identified by FTMap and DrugPy are the Thr14 (located in the interface of the L- and R-subdomains) and Arg106 (located in the L-subdomain) cavities.

**Figure 7:**
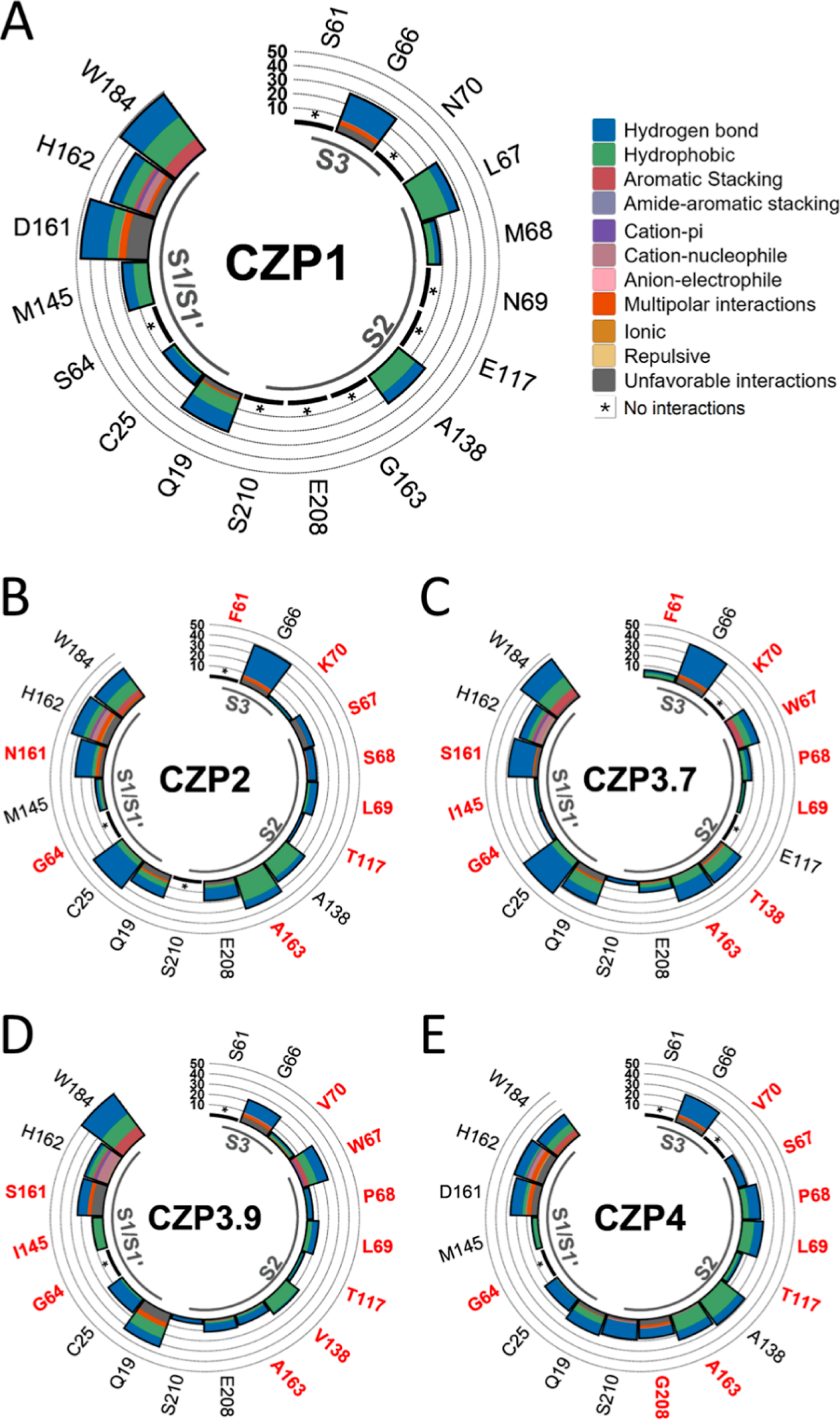
Profile of intermolecular interactions between virtual probes and active site residues for all subtypes using the representative structures from molecular dynamics clusters. Substitutions in Cruzipain sequences impact the interactions between the enzyme and the probes. We analyzed the residues from the subsites S3, S2, S1, and S1′ for CZP1 (A), CZP2 (B), CZP3.7 (C), CZP3.9 (D), and CZP4 (E). The colors of the bars correspond to the sort of interaction, as stated in (A), and their height is proportional to the interaction frequency. The residues in red are different from the CZP1 residue at the same position.

We also analyzed FTMap results with LUNA to investigate the impact of the residue substitutions in the interaction profile with the probes (Figures 7 and S6). The interactions predominantly consisted of hydrogen bonds and hydrophobic interactions across all subsites. The presence of the aromatic residue tryptophan at positions 67 (in CZP3.7 and CZP3.9) and 184 led to aromatic stacking interactions with the probes. Additional interactions, including amide–aromatic stacking, cation–pi, cation–nucleophile, anion–electrophile, multipolar, ionic, and repulsive forces, were also seen between the probes and the residues comprising the subsites, but with lower frequency.

Despite conserved residues like Gly66 and the catalytic Cys25 exhibiting over 30% interactions with probes across modeled structures in all subtypes (Figure S6), their interactions varied in the representative structures derived from the simulations (Figure 7). Gly66 primarily formed hydrogen bonds with FTMap probes, achieving a frequency of 20% in CZP3.9 (Figure 7D), whereas in other subtypes, the overall frequency was at least 30%, peaking at 40% in CZP2 (Figure 7B) and CZP3.7 (Figure 7C). Cys25 also mainly formed hydrogen bond interactions with the probes at a very low frequency in CZP1 (Figure 7A), CZP3.9 (Figure 7D), and CZP4 (Figure 7E) but reached over 30% in CZP2 (Figure 7B) and CZP3.7 (Figure 7C). This might be explained by the substitutions that impact the shape and polarity of the active site. The residue substitutions may have also affected other conserved residues, including Gln19, His162, and Trp184; however, their overall frequency and interaction types experienced fewer alterations compared to CZP1. Another noteworthy residue is Glu208, an important residue associated with substrate selectivity in cysteine proteases whose protonation state has been suggested to vary based on the group interacting with it.^58,59^ Its presence at the S2 subsite underscores the accessibility of this subsite to the probes. In both the representative structures from the simulations (Figure 7) and the modeled structures (Figure S6), interactions between Glu208 and the probes used by FTMap were minimal (under 30%) or nonexistent. Interestingly, Gly at this position in CZP4 exhibited interactions with the probes at a frequency of around 20%, the second highest after CZP2.

The substitutions at positions 61 and 70 in the S3 subsite did not affect the interaction with the probes since the only interacting residue was Gly66, which is conserved in all subtypes (Figure 7A–E). There is only one exception, CZP3.7 (Figure 7C). In this case, the replacement of Phe61 resulted in a few infrequent interactions involving hydrogen bonds, hydrophobic interactions, and cation–pi interactions. Interestingly, neither Ser61 nor Asn70 forms any interactions with the ligands that are cocrystallized with cruzain.^33,41^

The S2 subsite, which had the highest number of substitutions, exhibited a diverse range of interactions with the probes. The residues at positions 138 and 163 were involved in hydrophobic and hydrogen bond interactions in all subtypes (Figure 7A–E), albeit with varying frequency. The presence of Trp67 in CZP3.7 (Figure 7C) and CZP3.9 (Figure 7D) facilitates the interaction of the probes by aromatic stacking and pi-stacking, as well as hydrophobic interactions and hydrogen bonding. The Ser67 substitution did not result in a higher frequency of interactions for CZP4 (Figure 7E) or CZP2 (Figure 7B), possibly due to the dynamics in the S2 “gate” mechanism previously mentioned for these subtypes. In the CZP4 subtype, it is noteworthy that all S2 subsite residues interacted with the probes at a frequency of at least 5%, mainly through hydrophobic interactions and hydrogen bonds. This suggests that the continuous open condition of the S2 subsite over the whole simulation time benefited the probe’s positioning.

The retention of Gln19, Cys25, His162, and Trp184 in the S1 and S1′ subsites led to the conservation and, in some cases, the enhancement of the frequency of interactions in CZP2, CZP3.7, CZP3.9, and CZP4, compared to CZP1 (Figure 7). The substitution Ser64Gly did not have a significant influence on probe interactions. However, residues at 161, which are positioned at the interface between the S2/S1′ subsites, and residues at 145 exhibit distinct interactions among the subtypes when probed. Residues Asp161 and Met145 in CZP1 are known to play a crucial role in recognition in S1’.^47^ Therefore, the presence of substitution in these positions leading to differential interactions between probes suggests that ligands might be recognized differently by the subtypes. While these results should not be overinterpreted, especially considering the limited accuracy of probe positioning by FTMap, our analysis indicates an impact of the substitutions on the patterns of interactions between the probes and Cruzipain subtypes.

## 3 Conclusions

Our computational comparative analysis of five Cruzipain from different subtypes demonstrates a complex landscape of structural variation and its possible effects on enzyme activity. Although the general structural similarity remains remarkably consistent, the subtle differences in the active site regions during the MD simulations highlight the complex evolutionary adaptations of these proteases. Significant differences were noted in the S2 subsite, which is essential for substrate specificity, ligand selectivity, and recognition. Each subtype exhibited structural alterations distinct from those of CZP1: CZP2 has an expanded subsite with modified polarity, CZP3.7 and CZP3.9 reveal partial subsite occlusion, and CZP4 shows considerable enlargement accompanied by a potentially major conformational shift involving the residue at position 208. In contrast, the S1/S1′ subsites exhibited notable conservation, suggesting a significant evolutionary restriction to preserve the enzyme’s essential catalytic activity.

Our findings offer insights into the structural diversity of Cruzipain subtypes and suggest opportunities for further exploration of the functional significance of these complex structural variations, especially through complementary experimental validation. Important questions remain, including a more comprehensive understanding of the expression profiles of each Cruzipain subtype and their biological roles for different *T. cruzi* strains. Still, the insights reported here may be essential for developing targeted therapies, especially in circumstances where Cruzipain is significantly involved, such as in parasite infections or disease mechanisms.

## 4 Methods

### 4.1 Structure Preparation

For the catalytic domain structure of cruzain (CZP1), the PDB ID 1ME3(60) was used, while CZP2, CZP3.7, CZP3.9, and CZP4 catalytic domains were previously obtained through comparative modeling in Santos et al.^41^ Water molecules, ligands, and other cofactors were removed. Hydrogen atoms were added using the H++ server,^61^ considering the protonation state of the amino acid residues at pH 5.5 (optimal pH for cruzain activity, employed in biochemical assays). The catalytic dyad protonation state was set accordingly to catalysis: deprotonated catalytic cysteine with a negative charge (Cys25) and positively charged catalytic histidine (His162). The Glu208 on the S2 subsite of CZP1, CZP2, CZP3.7, and CZP3.9 was considered deprotonated with a negative charge.

Structural superposition of the protein’s Cα atoms and the electrostatic charges of the residues were performed with UCSF ChimeraX^62^ and APBS program,^63^ respectively.

### 4.2 Principal Component Analysis of Experimental Structures

The ensemble of Cruzipain experimental conformations was obtained using ProDy v.2.0.^64^ First, we carried out a BLAST search against the PDB to retrieve structures sharing at least 85% sequence identity with the reference structure 1ME3, leading to the recall of 31 cruzain PDB structures. Then, the C_α_ atoms from these structures were superposed using the Kabsch algorithm. The most relevant structural variations found in the data set were identified with a principal component analysis (PCA), which is based on the diagonalization of the covariance matrix, *C*_(i,j)_, of atomic positions whose elements are represented by
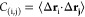
where Δ**r**_i_ and Δ**r**_j_ indicate the displacement vectors of atoms i and j, respectively, from their average positions, and brackets indicate ensemble averages. Then, an eigenvalue problem was solved, resulting in 3N PCs that were sorted according to their fractional contributions to the overall variance.

We used an in-house R script to calculate the free energy landscapes. The free energy difference (Δ*G*_α_) of a particular state α with respect to the most populated one (taken as reference) was calculated according to the probability of finding these states as given by
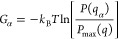
where *k*_B_ is the Boltzmann constant, *T* is the temperature of the simulations (310 K), and *P*(*q*_α_) is an estimate of the probability density function obtained from bidimensional kernel density estimates of the projections onto the first two principal components calculated over the concatenated standard MD trajectories. *P*_max_(*q*) is the probability of the most visited state.

### 4.3 Molecular Dynamics Simulations

The structure of the catalytic domain of each subtype was prepared as input for simulations by using the same protocol. Atomic partial charges were taken from the AMBER FF14SB^65^ force field and computed with the LEaP module of AmberTools19.^66^ The disulfide bonds observed in crystallographic structures of cruzain, between Cys22 and Cys63, Cys155 and Cys203, and Cys56 and Cys101, were set for all structures.

The structures were enclosed within an octahedral box filled with TIP3P water molecules.^67^ Box dimensions were set to include 11 Å of the outermost protein atoms in all Cartesian directions. Additionally, 8 Na^+^ ions were introduced to neutralize the system. Simulations were performed using the pmemd.cuda program from Amber 18.^66^ Electrostatic interactions were handled using the Particle-Mesh Ewald algorithm^68^ with a cutoff of 10 Å.

The energy minimization and relaxation procedures were performed using the same protocol described in our previous studies.^69,70^ Three independent production simulations of 300 ns each were performed in the *NPT* ensemble. Temperature and pressure control were kept constant at 310 K and 1 atm, respectively. The Langevin thermostat^71^ with a collision frequency of 2 ps^–1^ was used for temperature control, and pressure coupling was performed using isotropic position scaling. Periodic boundary conditions and the SHAKE algorithm^72^ were employed, allowing a 2 fs time step for the simulations. The trajectories were created by saving the frames every 20 ps of simulation, that is, every 10,000 steps.

### 4.4 Structural and Trajectory Analyses

VMD^73^ was used to calculate root-mean-square deviations (RMSD), root-mean-square fluctuations (RMSF), and interatomic distances. For RMSD, only the Cα atoms of residues 2 to 211 were considered. The remaining residues (1 and 213–215) were not considered as they presented much higher flexibility, as commonly observed for N- and C-terminal residues. Similarly, for RMSF, the overlapping of the side chains between residues 2 and 211 was considered. For the interatomic distances, the simple arithmetic average between the side chain atoms of residues at positions 67 and 208 of each subtype was measured. These residues are known to open or close a pocket beyond the S2 subsite of the protease.^56^

Cluster analysis was performed with the CPPTRAJ program^74^ of AmberTools19^66^ using a cutoff of 1.0 Å, considering all α-carbon atoms from the last 50 ns of each replicate simulation for all subtypes.

All three-dimensional visualizations were done using UCSF ChimeraX,^62^ and all plots were created using R^75^ and R Studio.^76^

### 4.5 Calculation of Active Site Volume

Approximately 1500 random frames from the trajectories were chosen to map the active site cavity using the MDpocket tool.^55^ MDpocket utilizes the pocket detection program Fpocket,^77^ which extensively uses Voronoi tessellation for cavity identification. The program offers data on the flexibility of pockets based on the frequency and density maps derived from the set of structures. To capture the full extent of the active site, we selected all grid points from the generated pocket frequency map that have a grid value of 0.1 or greater.

### 4.6 Correlation Network Analysis

The cross-correlation and network analyses were conducted using the R Bio3D package.^78^ The final 50 ns of each replicate simulation of each subtype were used to obtain the correlation matrix and to produce dynamic cross-correlation maps . Through the correlation maps, the cross-correlation coefficients were evaluated and allowed the determination of correlated or anticorrelated regions of the subtypes. Networks were constructed from the correlation data computed using atoms whose close contact was up to 4 Å apart during at least 75% of the simulation. In these networks, the residues are grouped by nodes that are connected by weights and edges, proportional to their degree of correlated movement.

Additionally, a measure of centrality known as betweenness centrality was computed to assess the relative significance of communication between each node (protein residue). This measure considers a node to be relevant if it is present in many of the shortest communication pathways that connect nodes (edges) across the whole network.^79^

### 4.7 Mapping of Subsites with Virtual Probes—FTMap

Representative structures of each of the three most populous molecular dynamics clusters, for each Cruzipain system or subtype, were submitted on the FTMap server.^80^ For molecular anchoring, 16 possible probe types, with different physicochemical properties, were kept. The results obtained were extracted and visually analyzed in PyMOL^81^ and loaded through the plugin DRUGpy. DRUGpy builds combinations of hotspots predicted by FTMap and classifies them as druggable (D), druggable small (Ds), borderline (B), or borderline small (Bs).^57^ Finally, we used LUNA’s^82^ default FTMap parser to recognize probe clusters from its output PDB files and default parameters to calculate protein–ligand interactions considering each probe. Interaction frequencies were then aggregated and calculated by type and by the probe cluster.

## Data Availability

The data underlying this study are openly available on zenodo.org at 10.5281/zenodo.14914661. We have used the following software for this article: UCSF Chimera version 1.15; APBS 3.0.0; R version 4.3.1 on RStudio version 2023.09.1; AMBER 18; CPPTRAJ version 4.14.0; VMD version 1.9.3; ProDy v.2.0; H++ server; FTMap server, Fpocket version 4.0 (MDpocket); PyMOL version 2.3.3; LUNA version 0.11.4; DRUGpy version 1.0; and Bio3D version 2.4.2.

## Supporting Information Available

Additional data concerning MD simulation results, dynamical cross-correlation matrices, betweenness values, druggability analysis, and profile of intermolecular interactions with virtual probes (PDF)

## Abbreviations

CD: Chagas disease
DCCMs: dynamic cross-correlation matrices
MD: molecular dynamics
RMSD: root-mean-square deviation
RMSF: root-mean-square fluctuation
SIP: square inner product

## References

[ref1] World Health Organization. Chagas disease (also known as American trypanosomiasis). 2024, https://www.who.int/news-room/fact-sheets/detail/chagas-disease-american-trypanosomiasis (accessed Oct 9, 2024).

[ref2] Pérez-Molina J. A.; Molina I. Chagas Disease. Lancet 2018, 391 (10115), 82–94. 10.1016/S0140-6736(17)31612-4. 28673423

[ref3] Imai K.; Misawa K.; Osa M.; Tarumoto N.; Sakai J.; Mikita K.; Sayama Y.; Fujikura Y.; Kawana A.; Murakami T.; Maesaki S.; Miura S.; Maeda T. Chagas Disease: A Report of 17 Suspected Cases in Japan, 2012–2017. Trop. Med. Health 2019, 47 (1), 38 10.1186/s41182-019-0168-3. 31223270 PMC6567435

[ref4] Castillo-Riquelme M. Chagas Disease in Non-Endemic Countries. Lancet Global Health 2017, 5 (4), e379–e380. 10.1016/S2214-109X(17)30090-6. 28256341

[ref5] Hotez P. J. The Rise of Neglected Tropical Diseases in the New Texas. PLoS Neglected Trop. Dis. 2018, 12 (1), e0005581 10.1371/journal.pntd.0005581. PMC5773009 29346369

[ref6] Irish A.; Whitman J. D.; Clark E. H.; Marcus R.; Bern C. Updated Estimates and Mapping for Prevalence of Chagas Disease among Adults, United States. Emerg. Infect. Dis. 2022, 28 (7), 1313 10.3201/eid2807.212221. PMC9239882 35731040

[ref7] Epstein D.; Nusser N.; Oliel S. 70% of people with Chagas don’t know they’re infected. 2024, https://www.paho.org/en/news/13-4-2021-70-people-chagas-dont-know-theyre-infected (accessed Oct 15, 2024).

[ref8] Junior P. A. S.; Molina I.; Murta S. M. F.; Sánchez-Montalvá A.; Salvador F.; Corrêa-Oliveira R.; Carneiro C. M. Experimental and Clinical Treatment of Chagas Disease: A Review. Am. J. Trop. Med. Hyg. 2017, 97 (5), 1289 10.4269/ajtmh.16-0761. 29016289 PMC5817734

[ref9] Murta S. M. F.; Lemos Santana P. A.; Jacques Dit Lapierre T. J. W.; Penteado A. B.; El Hajje M.; Navarro Vinha T. C.; Liarte D. B.; De Souza M. L.; Goulart Trossini G. H.; De Oliveira Rezende Júnior C.; De Oliveira R. B.; Ferreira R. S. New Drug Discovery Strategies for the Treatment of Benznidazole-Resistance in *Trypanosoma Cruzi*, the Causative Agent of Chagas Disease. Expert Opin. Drug Discovery 2024, 19 (6), 741–753. 10.1080/17460441.2024.2349155. 38715393

[ref10] De Rycker M.; Wyllie S.; Horn D.; Read K. D.; Gilbert I. H. Anti-Trypanosomatid Drug Discovery: Progress and Challenges. Nat. Rev. Microbiol. 2023, 21 (1), 35–50. 10.1038/s41579-022-00777-y. 35995950 PMC9395782

[ref11] Laureano De Souza M.; Lapierre T. J. W. J. D.; Vitor De Lima Marques G.; Ferraz W. R.; Penteado A. B.; Henrique Goulart Trossini G.; Murta S. M. F.; De Oliveira R. B.; De Oliveira Rezende C.; Ferreira R. S. Molecular Targets for Chagas Disease: Validation, Challenges and Lead Compounds for Widely Exploited Targets. Expert Opin. Ther. Targets 2023, 27 (10), 911–925. 10.1080/14728222.2023.2264512. 37772733

[ref12] Murta A. C. M.; Persechini P. M.; Padron T. de S.; de Souza W.; Guimarães J. A.; Scharfstein J. Structural and Functional Identification of GP57/51 Antigen of Trypanosoma Cruzi as a Cysteine Proteinase. Mol. Biochem. Parasitol. 1990, 43 (1), 27–38. 10.1016/0166-6851(90)90127-8. 1705310

[ref13] Cazzulo J. J.; Cazzulo Franke M. C.; Martínez J.; Franke de Cazzulo B. M. Some Kinetic Properties of a Cysteine Proteinase (Cruzipain) from Trypanosoma Cruzi. Biochim. Biophys. Acta, Protein Struct. Mol. Enzymol. 1990, 1037 (2), 186–191. 10.1016/0167-4838(90)90166-D. 2407295

[ref14] Eakin A. E.; Mills A. A.; Harth G.; McKerrow J. H.; Craik C. S. The Sequence, Organization, and Expression of the Major Cysteine Protease (Cruzain) from Trypanosoma Cruzi. J. Biol. Chem. 1992, 267 (11), 7411–7420. 10.1016/S0021-9258(18)42533-1. 1559982

[ref15] Meirelles M. N. L.; Juliano L.; Carmona E.; Silva S. G.; Costa E. M.; Murta A. C. M.; Scharfstein J. Inhibitors of the Major Cysteinyl Proteinase (GP57/51) Impair Host Cell Invasion and Arrest the Intracellular Development of Trypanosoma Cruzi in Vitro. Mol. Biochem. Parasitol. 1992, 52 (2), 175–184. 10.1016/0166-6851(92)90050-T. 1620157

[ref16] Franke de Cazzulo B. M.; Martínez J.; North M. J.; Coombs G. H.; Cazzulo J.-J. Effects of Proteinase Inhibitors on the Growth and Differentiation of Trypanosoma Cruzi. FEMS Microbiol. Lett. 1994, 124 (1), 81–86. 10.1111/j.1574-6968.1994.tb07265.x. 8001773

[ref17] Tomas A. M.; Miles M. A.; Kelly J. M. Overexpression of Cruzipain, the Major Cysteine Proteinase of Trypanosoma Cruzi, Is Associated with Enhanced Metacyclogenesis. Eur. J. Biochem. 1997, 244 (2), 596–603. 10.1111/j.1432-1033.1997.t01-1-00596.x. 9119029

[ref18] Scharfstein J.; Schmitz V.; Morandi V.; Capella M. M. A.; Lima A. P. C. A.; Morrot A.; Juliano L.; Müller-Esterl W. Host Cell Invasion by TRYPANOSOMA cRUZI Is Potentiated by Activation of Bradykinin B2 Receptors. J. Exp. Med. 2000, 192 (9), 1289–1300. 10.1084/jem.192.9.1289. 11067878 PMC2193362

[ref19] Doyle P. S.; Zhou Y. M.; Hsieh I.; Greenbaum D. C.; McKerrow J. H.; Engel J. C. The Trypanosoma Cruzi Protease Cruzain Mediates Immune Evasion. PLoS Pathog. 2011, 7 (9), e1002139 10.1371/journal.ppat.1002139. 21909255 PMC3164631

[ref20] Schmitz V.; Almeida L. N.; Svensjö E.; Monteiro A. C.; Köhl J.; Scharfstein J. C5a and Bradykinin Receptor Cross-Talk Regulates Innate and Adaptive Immunity in Trypanosoma Cruzi Infection. J. Immunol. 2014, 193 (7), 3613–3623. 10.4049/jimmunol.1302417. 25187655

[ref21] Andrade D.; Serra R.; Svensjö E.; Lima A. P. C.; Ramos Junior E. S.; Fortes F. S.; Morandini A. C. F.; Morandi V.; Soeiro M. de N.; Tanowitz H. B.; Scharfstein J. Trypanosoma Cruzi Invades Host Cells through the Activation of Endothelin and Bradykinin Receptors: A Converging Pathway Leading to Chagasic Vasculopathy. Br. J. Pharmacol. 2012, 165 (5), 1333–1347. 10.1111/j.1476-5381.2011.01609.x. 21797847 PMC3372720

[ref22] Rocha D. A.; Silva E. B.; Fortes I. S.; Lopes M. S.; Ferreira R. S.; Andrade S. F. Synthesis and Structure-Activity Relationship Studies of Cruzain and Rhodesain Inhibitors. Eur. J. Med. Chem. 2018, 157, 1426–1459. 10.1016/j.ejmech.2018.08.079. 30282318

[ref23] McKerrow J. H. Update on Drug Development Targeting Parasite Cysteine Proteases. PLoS Neglected Trop. Dis. 2018, 12 (8), e0005850 10.1371/journal.pntd.0005850. PMC6107105 30138309

[ref24] Prates J. L. B.; Lopes J. R.; Chin C. M.; Ferreira E. I.; Dos Santos J. L.; Scarim C. B. Discovery of Novel Inhibitors of Cruzain Cysteine Protease of Trypanosoma Cruzi. Curr. Med. Chem. 2024, 31 (16), 2285–2308. 10.2174/0109298673254864230921090519. 37888814

[ref25] Caffrey C. R.; Steverding D.; Ferreira R. S.; De Oliveira R. B.; O’Donoghue A. J.; Monti L.; Ballatore C.; Bachovchin K. A.; Ferrins L.; Pollastri M. P.; Zorn K. M.; Foil D. H.; Clark A. M.; Mottin M.; Andrade C. H.; De Siqueira-Neto J. L.; Ekins S. Drug Discovery and Development for Kinetoplastid Diseases. In Burger’s Medicinal Chemistry and Drug Discovery; Wiley, 2021; pp 1–79.

[ref26] Alves L.; Santos D. A.; Cendron R.; Rocho F. R.; Matos T. K. B.; Leitão A.; Montanari C. A. Nitrile-Based Peptoids as Cysteine Protease Inhibitors. Bioorg. Med. Chem. 2021, 41, 116211 10.1016/j.bmc.2021.116211. 33991733

[ref27] Barbosa Da Silva E.; Rocha D. A.; Fortes I. S.; Yang W.; Monti L.; Siqueira-Neto J. L.; Caffrey C. R.; McKerrow J.; Andrade S. F.; Ferreira R. S. Structure-Based Optimization of Quinazolines as Cruzain and *Tbr* CATL Inhibitors. J. Med. Chem. 2021, 64 (17), 13054–13071. 10.1021/acs.jmedchem.1c01151. 34461718

[ref28] Barbosa Da Silva E.; Sharma V.; Hernandez-Alvarez L.; Tang A. H.; Stoye A.; O’Donoghue A. J.; Gerwick W. H.; Payne R. J.; McKerrow J. H.; Podust L. M. Intramolecular Interactions Enhance the Potency of Gallinamide A Analogues against *Trypanosoma Cruzi*. J. Med. Chem. 2022, 65 (5), 4255–4269. 10.1021/acs.jmedchem.1c02063. 35188371

[ref29] Santos V. C.; Ferreira R. S. Computational Approaches towards the Discovery and Optimisation of Cruzain Inhibitors. Mem. Inst. Oswaldo Cruz 2022, 117, e210385 10.1590/0074-02760210385. 35293427 PMC8925305

[ref30] De Oliveira A. S.; Valli M.; Ferreira L. L.; Souza J. M.; Krogh R.; Meier L.; Abreu H. R.; Voltolini B. G.; Llanes L. C.; Nunes R. J.; Braga A. L.; Andricopulo A. D. Novel Trypanocidal Thiophen-Chalcone Cruzain Inhibitors: Structure- and Ligand-Based Studies. Future Med. Chem. 2022, 14 (11), 795–808. 10.4155/fmc-2022-0013. 35543430

[ref31] Jasinski G.; Salas-Sarduy E.; Vega D.; Fabian L.; Florencia Martini M.; Moglioni A. G. Design, Synthesis and Biological Evaluation of Novel Thiosemicarbazones as Cruzipain Inhibitors. Eur. J. Med. Chem. 2023, 254, 115345 10.1016/j.ejmech.2023.115345. 37054562

[ref32] Do Valle Moreira T.; Martins L. C.; Diniz L. A.; Bernardes T. C. D.; De Oliveira R. B.; Ferreira R. S. Screening the Pathogen Box to Discover and Characterize New Cruzain and TbrCatL Inhibitors. Pathogens 2023, 12 (2), 251 10.3390/pathogens12020251. 36839523 PMC9967275

[ref33] Santos V. C.; Leite P. G.; Santos L. H.; Pascutti P. G.; Kolb P.; Machado F. S.; Ferreira R. S. Structure-Based Discovery of Novel Cruzain Inhibitors with Distinct Trypanocidal Activity Profiles. Eur. J. Med. Chem. 2023, 257, 115498 10.1016/j.ejmech.2023.115498. 37290182

[ref34] Cerutti J. P.; Diniz L. A.; Santos V. C.; Vilchez Larrea S. C.; Alonso G. D.; Ferreira R. S.; Dehaen W.; Quevedo M. A. Structure-Aided Computational Design of Triazole-Based Targeted Covalent Inhibitors of Cruzipain. Molecules 2024, 29 (17), 4224 10.3390/molecules29174224. 39275072 PMC11396839

[ref35] Engel J. C.; Doyle P. S.; Hsieh I.; McKerrow J. H. Cysteine Protease Inhibitors Cure an Experimental Trypanosoma Cruzi Infection. J. Exp. Med. 1998, 188 (4), 725–734. 10.1084/jem.188.4.725. 9705954 PMC2213346

[ref36] Barr S. C.; Warner K. L.; Kornreic B. G.; Piscitelli J.; Wolfe A.; Benet L.; McKerrow J. H. A Cysteine Protease Inhibitor Protects Dogs from Cardiac Damage during Infection by Trypanosoma Cruzi. Antimicrob. Agents Chemother. 2005, 49 (12), 5160–5161. 10.1128/AAC.49.12.5160-5161.2005. 16304193 PMC1315979

[ref37] Pauli I.; Rezende C. D. O. Jr.; Slafer B. W.; Dessoy M. A.; De Souza M. L.; Ferreira L. L. G.; Adjanohun A. L. M.; Ferreira R. S.; Magalhães L. G.; Krogh R.; Michelan-Duarte S.; Del Pintor R. V.; Da Silva F. B. R.; Cruz F. C.; Dias L. C.; Andricopulo A. D. Multiparameter Optimization of Trypanocidal Cruzain Inhibitors With In Vivo Activity and Favorable Pharmacokinetics. Front. Pharmacol 2022, 12, 774069 10.3389/fphar.2021.774069. 35069198 PMC8767159

[ref38] Ferreira R. A.; Pauli I.; Sampaio T. S.; de Souza M. L.; Ferreira L. L.; Magalhaes L. G.; Rezende C. d. O. Jr; Ferreira R. S.; Krogh R.; Dias L. C.; et al. Structure-Based and Molecular Modeling Studies for the Discovery of Cyclic Imides as Reversible Cruzain Inhibitors with Potent Anti-Trypanosoma Cruzi Activity. Front. Chem. 2019, 7, 798 10.3389/fchem.2019.00798. 31824926 PMC6886403

[ref39] de Souza M. L.; de Oliveira Rezende Junior C.; Ferreira R. S.; Espinoza Chavez R. M.; Ferreira L. L.; Slafer B. W.; Magalhaes L. G.; Krogh R.; Oliva G.; Cruz F. C.; et al. Discovery of Potent, Reversible, and Competitive Cruzain Inhibitors with Trypanocidal Activity: A Structure-Based Drug Design Approach. J. Chem. Inf. Model. 2019, 60 (2), 1028–1041. 10.1021/acs.jcim.9b00802. 31765144

[ref40] Mattos E. C.; Canuto G.; Manchola N. C.; Magalhães R. D. M.; Crozier T. W. M.; Lamont D. J.; Tavares M. F. M.; Colli W.; Ferguson M. A. J.; Alves M. J. M. Reprogramming of Trypanosoma Cruzi Metabolism Triggered by Parasite Interaction with the Host Cell Extracellular Matrix. PLoS Neglected Trop. Dis. 2019, 13 (2), e0007103 10.1371/journal.pntd.0007103. PMC6380580 30726203

[ref41] Santos V. C.; Oliveira A. E. R.; Campos A. C. B.; Reis-Cunha J. L.; Bartholomeu D. C.; Teixeira S. M. R.; Lima A. P. C. A.; Ferreira R. S. The Gene Repertoire of the Main Cysteine Protease of Trypanosoma Cruzi, Cruzipain, Reveals Four Sub-Types with Distinct Active Sites. Sci. Rep. 2021, 11 (1), 18231 10.1038/s41598-021-97490-2. 34521898 PMC8440672

[ref42] Lima A.P.; Tessier D.; Thomas D.; Scharfstein J.; Storer A.; Vernet T. Identification of New Cysteine Protease Gene Isoforms in Trypanosoma Cruzi. Mol. Biochem. Parasitol. 1994, 67 (2), 333–338. 10.1016/0166-6851(94)00144-8. 7870137

[ref43] Lima A. P. C. A.; dos Reis F. C. G.; Serveau C.; Lalmanach G.; Juliano L.; Ménard R.; Vernet T.; Thomas D. Y.; Storer A. C.; Scharfstein J. Cysteine Protease Isoforms from Trypanosoma Cruzi, Cruzipain 2 and Cruzain, Present Different Substrate Preference and Susceptibility to Inhibitors. Mol. Biochem. Parasitol. 2001, 114 (1), 41–52. 10.1016/S0166-6851(01)00236-5. 11356512

[ref44] Lima A. P. C. A.; Almeida P. C.; Tersariol I. L. S.; Schmitz V.; Schmaier A. H.; Juliano L.; Hirata I. Y.; Müller-Esterl W.; Chagas J. R.; Scharfstein J. Heparan Sulfate Modulates Kinin Release by Trypanosoma Cruzi through the Activity of Cruzipain. J. Biol. Chem. 2002, 277 (8), 5875–5881. 10.1074/jbc.M108518200. 11726662

[ref45] dos Reis F. C. G.; Júdice W. A. S.; Juliano M. A.; Juliano L.; Scharfstein J.; De A.; Lima A. P. C. The Substrate Specificity of Cruzipain 2, a Cysteine Protease Isoform from Trypanosoma Cruzi. FEMS Microbiol. Lett. 2006, 259 (2), 215–220. 10.1111/j.1574-6968.2006.00267.x. 16734782

[ref46] Gillmor S. A.; Craik C. S.; Fletterick R. J. Structural Determinants of Specificity in the Cysteine Protease Cruzain. Protein Sci. 1997, 6 (8), 1603–1611. 10.1002/pro.5560060801. 9260273 PMC2143760

[ref47] Brinen L. S.; Hansell E.; Cheng J.; Roush W. R.; McKerrow J. H.; Fletterick R. J. A Target within the Target: Probing Cruzain’s P1′ Site to Define Structural Determinants for the Chagas’ Disease Protease. Structure 2000, 8 (8), 831–840. 10.1016/S0969-2126(00)00173-8. 10997902

[ref48] Martins L. C.; Torres P. H. M.; De Oliveira R. B.; Pascutti P. G.; Cino E. A.; Ferreira R. S. Investigation of the Binding Mode of a Novel Cruzain Inhibitor by Docking, Molecular Dynamics, Ab Initio and MM/PBSA Calculations. J. Comput. Aided Mol. Des. 2018, 32 (5), 591–605. 10.1007/s10822-018-0112-3. 29564808

[ref49] Santos L. H.; Waldner B. J.; Fuchs J. E.; Pereira G. A. N.; Liedl K. R.; Caffarena E. R.; Ferreira R. S. Understanding Structure–Activity Relationships for Trypanosomal Cysteine Protease Inhibitors by Simulations and Free Energy Calculations. J. Chem. Inf. Model. 2019, 59 (1), 137–148. 10.1021/acs.jcim.8b00557. 30532974

[ref50] Toman N. P.; Kamenik A. S.; Santos L. H.; Hofer F.; Liedl K. R.; Ferreira R. S. Profiling Selectivity of Chagasin Mutants towards Cysteine Proteases Cruzain or Cathepsin L through Molecular Dynamics Simulations. J. Biomol. Struct. Dyn. 2021, 39 (16), 5940–5952. 10.1080/07391102.2020.1796797. 32715978

[ref51] Barbosa Da Silva E.; Dall E.; Briza P.; Brandstetter H.; Ferreira R. S. Cruzain Structures: Apocruzain and Cruzain Bound to *S* -Methyl Thiomethanesulfonate and Implications for Drug Design. Acta Crystallogr., Sect. F:Struct. Biol. Commun. 2019, 75 (6), 419–427. 10.1107/S2053230X19006320. 31204688 PMC6572096

[ref52] Hernández Alvarez L.; Barreto Gomes D. E.; Hernández González J. E.; Pascutti P. G. Dissecting a Novel Allosteric Mechanism of Cruzain: A Computer-Aided Approach. PLoS One 2019, 14 (1), e0211227 10.1371/journal.pone.0211227. 30682119 PMC6347273

[ref53] Schechter I.; Berger A. On the Size of the Active Site in Proteases. I. Papain. Biochem. Biophys. Res. Commun. 1967, 27 (2), 157–162. 10.1016/S0006-291X(67)80055-X. 6035483

[ref54] Novinec M.; Lenarčič B.; Turk B. Cysteine Cathepsin Activity Regulation by Glycosaminoglycans. BioMed. Res. Int. 2014, 2014, 1–9. 10.1155/2014/309718. PMC4283429 25587532

[ref55] Schmidtke P.; Bidon-Chanal A.; Luque F. J.; Barril X. MDpocket: Open-Source Cavity Detection and Characterization on Molecular Dynamics Trajectories. Bioinformatics 2011, 27 (23), 3276–3285. 10.1093/bioinformatics/btr550. 21967761

[ref56] Durrant J. D.; Keränen H.; Wilson B. A.; McCammon J. A. Computational Identification of Uncharacterized Cruzain Binding Sites. PLoS Neglected Trop. Dis. 2010, 4 (5), e676 10.1371/journal.pntd.0000676. PMC2867933 20485483

[ref57] Teixeira O.; Lacerda P.; Froes T. Q.; Nonato M. C.; Castilho M. S. Druggable Hot Spots in Trypanothione Reductase: Novel Insights and Opportunities for Drug Discovery Revealed by DRUGpy. J. Comput. Aided Mol. Des. 2021, 35 (8), 871–882. 10.1007/s10822-021-00403-8. 34181199

[ref58] Zhai X.; Meek T. D. Catalytic Mechanism of Cruzain from *Trypanosoma Cruzi* As Determined from Solvent Kinetic Isotope Effects of Steady-State and Pre-Steady-State Kinetics. Biochemistry 2018, 57 (22), 3176–3190. 10.1021/acs.biochem.7b01250. 29336553 PMC10569748

[ref59] Santos V. C.; Campos A. C. B.; Waldner B. J.; Liedl K. R.; Ferreira R. S. Impact of Different Protonation States on Virtual Screening Performance against Cruzain. Chem. Biol. Drug Des. 2022, 99 (5), 703–716. 10.1111/cbdd.14008. 34923756

[ref60] Huang L.; Brinen L. S.; Ellman J. A. Crystal Structures of Reversible Ketone-Based Inhibitors of the Cysteine Protease Cruzain. Bioorg. Med. Chem. 2003, 11 (1), 21–29. 10.1016/S0968-0896(02)00427-3. 12467703

[ref61] Anandakrishnan R.; Aguilar B.; Onufriev A. V. H++ 3.0: Automating pK Prediction and the Preparation of Biomolecular Structures for Atomistic Molecular Modeling and Simulations. Nucleic Acids Res. 2012, 40 (W1), W537–W541. 10.1093/nar/gks375. 22570416 PMC3394296

[ref62] Meng E. C.; Goddard T. D.; Pettersen E. F.; Couch G. S.; Pearson Z. J.; Morris J. H.; Ferrin T. E. UCSF ChimeraX: Tools for Structure Building and Analysis. Protein Sci. 2023, 32 (11), e4792 10.1002/pro.4792. 37774136 PMC10588335

[ref63] Jurrus E.; Engel D.; Star K.; Monson K.; Brandi J.; Felberg L. E.; Brookes D. H.; Wilson L.; Chen J.; Liles K.; Chun M.; Li P.; Gohara D. W.; Dolinsky T.; Konecny R.; Koes D. R.; Nielsen J. E.; Head-Gordon T.; Geng W.; Krasny R.; Wei G.; Holst M. J.; McCammon J. A.; Baker N. A. Improvements to the APBS Biomolecular Solvation Software Suite. Protein Sci. 2018, 27 (1), 112–128. 10.1002/pro.3280. 28836357 PMC5734301

[ref64] Bakan A.; Meireles L. M.; Bahar I. ProDy: Protein Dynamics Inferred from Theory and Experiments. Bioinformatics 2011, 27 (11), 1575–1577. 10.1093/bioinformatics/btr168. 21471012 PMC3102222

[ref65] Maier J. A.; Martinez C.; Kasavajhala K.; Wickstrom L.; Hauser K. E.; Simmerling C. ff14SB: Improving the Accuracy of Protein Side Chain and Backbone Parameters from ff99SB. J. Chem. Theory Comput. 2015, 11 (8), 3696–3713. 10.1021/acs.jctc.5b00255. 26574453 PMC4821407

[ref66] Case D. A.; Ben-Shalom I. Y.; Brozell S. R.; Cerutti D. S.; Cheatham T. E. I.; Cruzeiro V. W. D.; Darden T. A.; Duke R. E.; Ghoreishi D.; Giambasu G.; Giese T.; Gilson M. K.; Gohlke H.; Goetz A. W.; Greene D.; Harris R.; Homeyer N.; Huang P. A. Amber; Amber, 2019; Vol. 2019.

[ref67] Jorgensen W. L.; Chandrasekhar J.; Madura J. D.; Impey R. W.; Klein M. L. Comparison of Simple Potential Functions for Simulating Liquid Water. J. Chem. Phys. 1983, 79 (2), 926–935. 10.1063/1.445869.

[ref68] Darden T.; York D.; Pedersen L. Particle Mesh Ewald: An *N* ·log(*N*) Method for Ewald Sums in Large Systems. J. Chem. Phys. 1993, 98 (12), 10089–10092. 10.1063/1.464397.

[ref69] Ben Yekhlef R.; Felicori L.; SantosF B OliveiraFadhloun L. H. C. R.; Torabi E.; Shahbazzadeh D.; Pooshang Bagheri K.; Salgado Ferreira R.; Borchani L.; Salgado Ferreira R.; Borchani L. Antigenic and Substrate Preference Differences between Scorpion and Spider Dermonecrotic Toxins, a Comparative Investigation. Toxins 2020, 12 (10), 631 10.3390/toxins12100631. 33019554 PMC7601583

[ref70] Santos L. H.; Caffarena E. R.; Ferreira R. S. pH and Non-Covalent Ligand Binding Modulate Zika Virus NS2B/NS3 Protease Binding Site Residues: Discoveries from MD and Constant pH MD Simulations. J. Biomol. Struct. Dyn. 2022, 40 (20), 10359–10372. 10.1080/07391102.2021.1943528. 34180376

[ref71] Adelman S. A.; Doll J. D. Generalized Langevin Equation Approach for Atom/Solid-Surface Scattering: General Formulation for Classical Scattering off Harmonic Solids. J. Chem. Phys. 1976, 64 (6), 2375–2388. 10.1063/1.432526.

[ref72] Ryckaert J.-P.; Ciccotti G.; Berendsen H. J. C. Numerical Integration of the Cartesian Equations of Motion of a System with Constraints: Molecular Dynamics of n-Alkanes. J. Comput. Phys. 1977, 23 (3), 327–341. 10.1016/0021-9991(77)90098-5.

[ref73] Humphrey W.; Dalke A.; Schulten K. VMD: Visual Molecular Dynamics. J. Mol. Graph. 1996, 14 (1), 33–38. 10.1016/0263-7855(96)00018-5. 8744570

[ref74] Roe D. R.; Cheatham T. E. PTRAJ and CPPTRAJ: Software for Processing and Analysis of Molecular Dynamics Trajectory Data. J. Chem. Theory Comput. 2013, 9 (7), 3084–3095. 10.1021/ct400341p. 26583988

[ref75] R Core Team. R: A Language and Environment for Statistical Computing, 2020. https://www.R-project.org/.

[ref76] RStudio Team. RStudio: Integrated Development Environment for R, 2020. http://www.rstudio.com/.

[ref77] Le Guilloux V.; Schmidtke P.; Tuffery P. Fpocket: An Open Source Platform for Ligand Pocket Detection. BMC Bioinf. 2009, 10 (1), 168 10.1186/1471-2105-10-168. PMC2700099 19486540

[ref78] Grant B. J.; Rodrigues A. P. C.; ElSawy K. M.; McCammon J. A.; Caves L. S. D. Bio3d: An R Package for the Comparative Analysis of Protein Structures. Bioinformatics 2006, 22 (21), 2695–2696. 10.1093/bioinformatics/btl461. 16940322

[ref79] Kolaczyk E. D. Statistical Analysis of Network Data: Methods and Models; Springer Series in Statistics: Springer New York: New York, NY, 2009.

[ref80] Kozakov D.; Grove L. E.; Hall D. R.; Bohnuud T.; Mottarella S. E.; Luo L.; Xia B.; Beglov D.; Vajda S. The FTMap Family of Web Servers for Determining and Characterizing Ligand-Binding Hot Spots of Proteins. Nat. Protoc. 2015, 10 (5), 733–755. 10.1038/nprot.2015.043. 25855957 PMC4762777

[ref81] Schrödinger L. L. C. The PyMOL Molecular Graphics System; PyMOL, 2015.

[ref82] Fassio A. V.; Shub L.; Ponzoni L.; McKinley J.; O’Meara M. J.; Ferreira R. S.; Keiser M. J.; De Melo Minardi R. C. Prioritizing Virtual Screening with Interpretable Interaction Fingerprints. J. Chem. Inf. Model. 2022, 62 (18), 4300–4318. 10.1021/acs.jcim.2c00695. 36102784

