# Supplementary material for: Unraveling Common Patterns and Differences among Cruzipains through Molecular Dynamics Simulations and Structural Analyses
Source: ACS Omega. 2025 May 2;10(18):19115–28. doi: 10.1021/acsomega.5c01876 (PMC12079592; doi:10.1021/acsomega.5c01876)
Supplement: Supplementary file 1 — ao5c01876_si_001.pdf [file ao5c01876_si_001.pdf]

Supporting Information

Unraveling common patterns and differences among cruzipains through molecular dynamics simulations and structural analyses

Lucianna Helene S. Santos[a], *Augusto César Broilo Campos*[b]. *Viviane Corrêa Santos*[b‡], *Alexandre Victor Fassio*[c], *Maurício G.S. Costa*[d], *Rafaela Salgado Ferreira*[b*]

[a] Institut Pasteur de Montevideo, Mataojo 2020, 11400, Montevideo, Uruguay.

[b] Departamento de Bioquímica e Imunologia, Universidade Federal de Minas Gerais, Avenida Antônio Carlos 6627, Belo Horizonte, 31270-901, Minas Gerais, Brazil.

[c] Instituto de Física de São Carlos, Universidade de São Paulo, São Carlos, São Paulo 13563-120, Brazil.

[d] Programa de Computação Científica, Vice Presidência de Educação Informação e Comunicação, Fundação Oswaldo Cruz, 21040-900 Rio de Janeiro, Brasil.

Corresponding Author
*Rafaela S. Ferreira – rafaelasf@icb.ufmg.br

Present Addresses
‡Viviane Corrêa Santos - Department of Chemistry, Grand Valley State University, 1 Campus Drive, Allendale, MI 49401, USA.

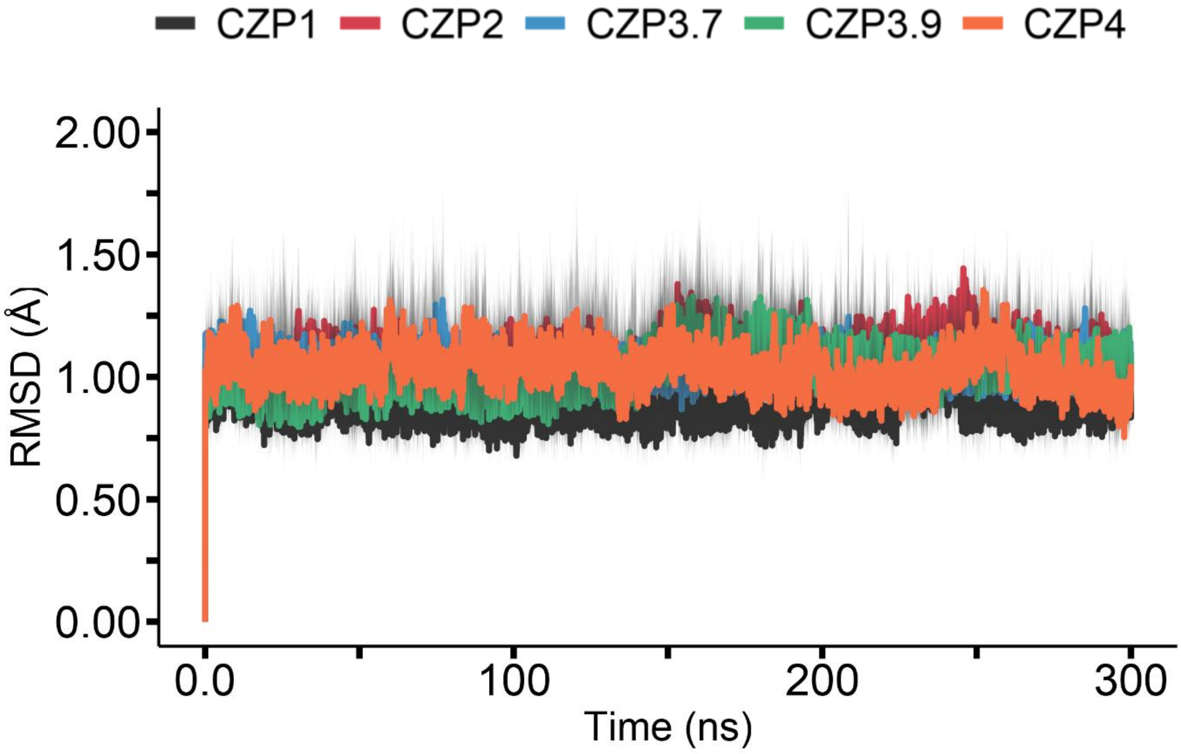

Figure S1. RMSD vs time for CZP1 (black), CZP2 (red), CZP3.7 (blue), CZP3.9 (green), and CZP4 (orange). Gray lines show deviations from all three trajectory replicas.

Table S1 – Correlations between flexibility calculated from MD simulations and experimental structures.

STRUCTURE	CORRELATION
**CZP1**	0.82
**CZP2**	0.78
**CZP3.7**	0.74
**CZP3.9**	0.65
**CZP4**	0.78

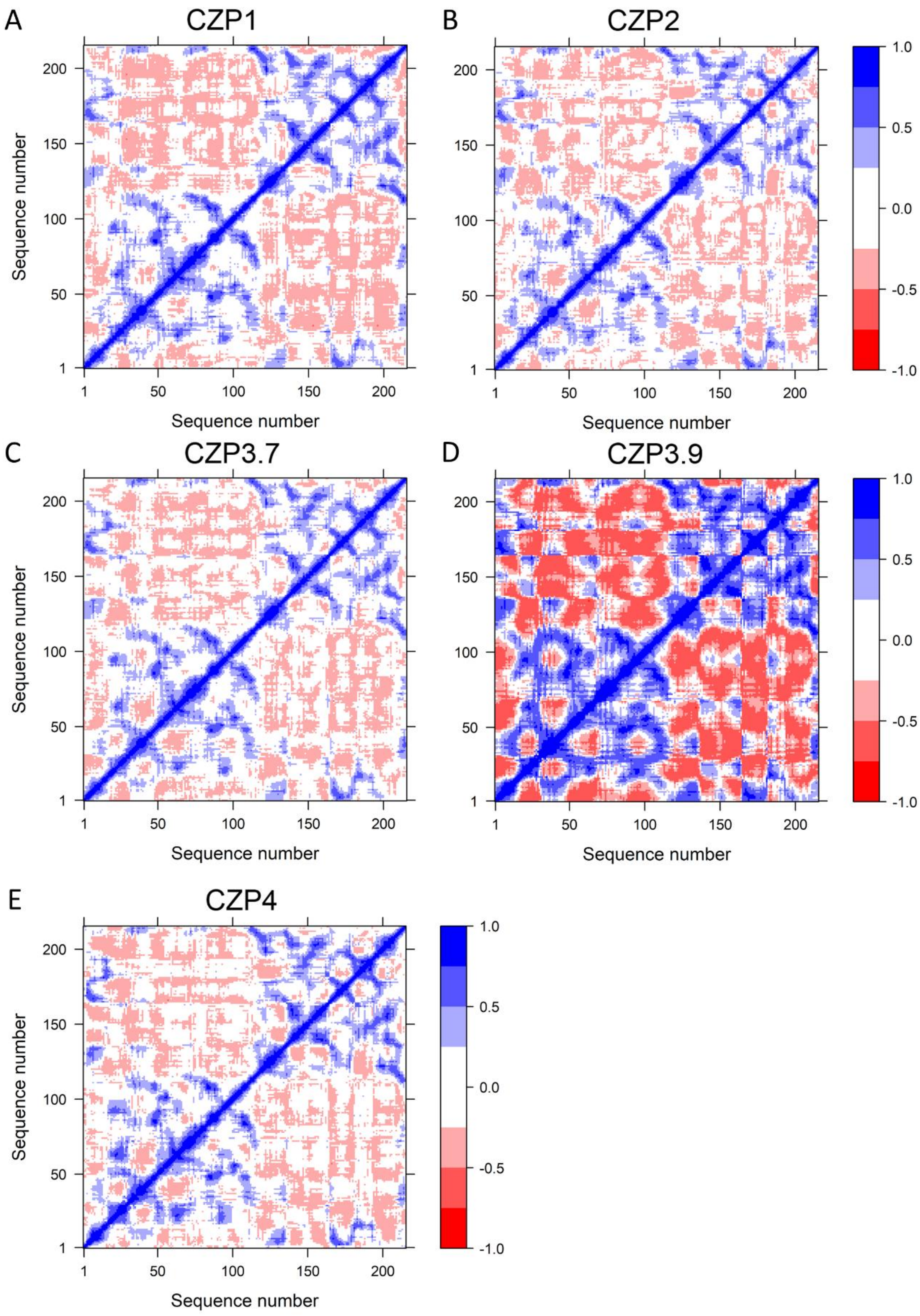

Figure S2. Maps of the dynamical cross-correlation matrixes (DCCM) for cruzipain subtypes. All three replicas for CZP1 (A), CZP2 (B), CZP3.7 (C), CZP3.9 (D), and CZP4 (E) were considered in the maps. Residues that exhibit positive correlation move in the same direction and are represented by blue regions on the map. In contrast, residues that show negative correlation move in opposite directions and are depicted by red regions. White areas of the maps indicate a lack of correlations.

Table S2 – Square inner product (SIP) among the betweenness profiles computed for all cruzipains.

	**CZP1**	**CZP2**	**CZP3.7**	**CZP3.9**	**CZP4**
**CZP1**	1.00	0.83	0.83	0.85	0.81
**CZP2**	0.83	1.00	0.77	0.83	0.75
**CZP3.7**	0.83	0.77	1.00	0.83	0.87
**CZP3.9**	0.85	0.83	0.83	1.00	0.80
**CZP4**	0.81	0.75	0.87	0.80	1.00

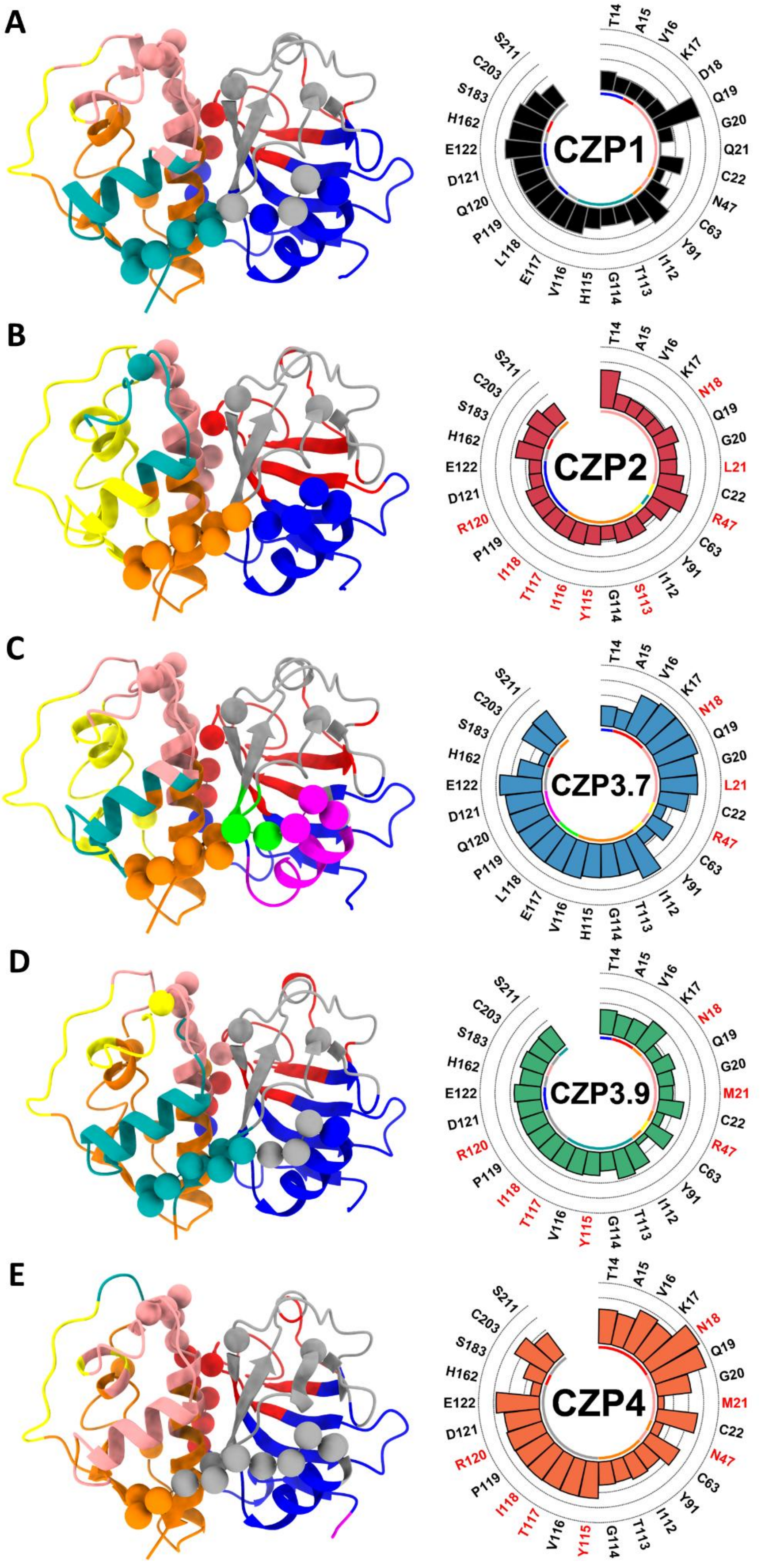
A
CZP1
T14
A15
V16
K17
D18
Q19
G20
Q21
C22
N47
C63
Y91
I112
T113
G114
H115
V116
E117
L118
P119
Q120
D121
E122
H162
S183
C203
S211
B
CZP2
N18
L21
R47
S113
Y115
I116
T117
I118
R120
C
CZP3.7
D
CZP3.9
M21
E
CZP4

Figure S3. Residue betweenness values across cruzipain subtypes using a filter of betweenness > 0.1. A) Residues with high betweenness from the CZP1 subtype were Gln19, Tyr91, Glu122, His162, and Ser183. B) Residues with high betweenness from the CZP2 subtype were His162, Thr14, and Arg47. C) Residues with high betweenness from the CZP3.7 subtype were Val16, Lys17, Asn18, Gln19, Gly20, Leu21, Ile112, Thr113, Gly114, His115, Val116, Glu117, Leu118, Pro119, Gln120, Asp121, Glu122, Cys203, and Ser211. D) Residues with high betweenness from the CZP3.9 subtype were Lys17, Thr113, and Glu122. E) Residues with high betweenness from the CZP4 subtype were Thr14, Ala15, Val16, Lys17, Asn18, Gln19, Gly20, Cys22, Cys63, Tyr115, Val116, Thr117, Ile118, Pro119, Arg120, Asp121, Glu122, and Cys203.

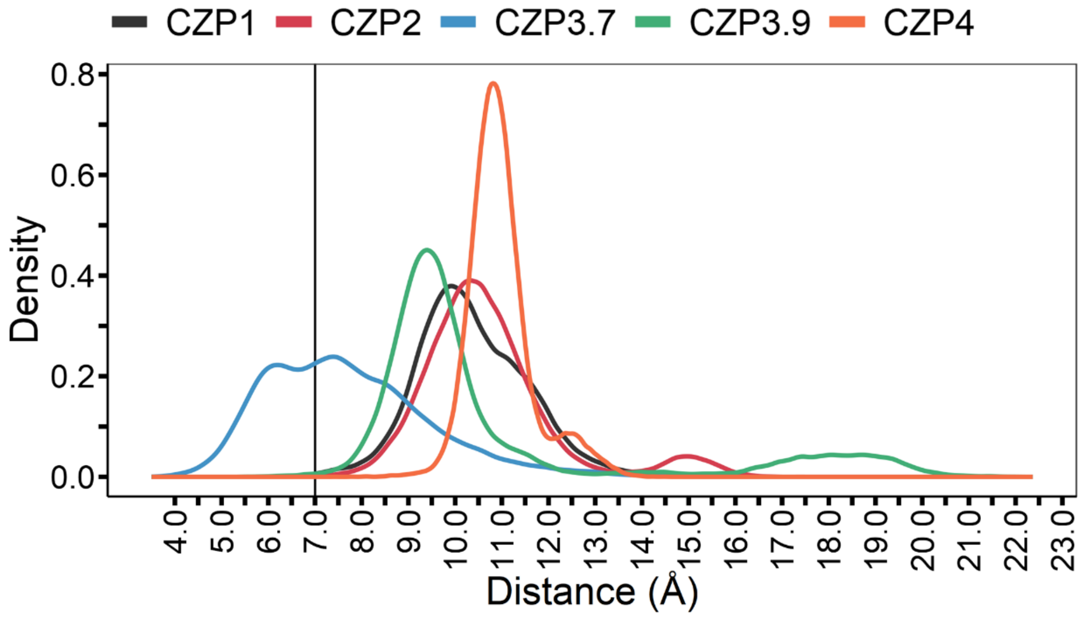

Figure S4. **The inter-residue distance between residues located at positions 61 and 67 measured during the MD simulations.** The CZP3.7 distance between Phe61 and Trp67 is the minimal distance among all pairings of residues 61 and 67, leading them to constant interact in possible aromatic interaction between the rings.

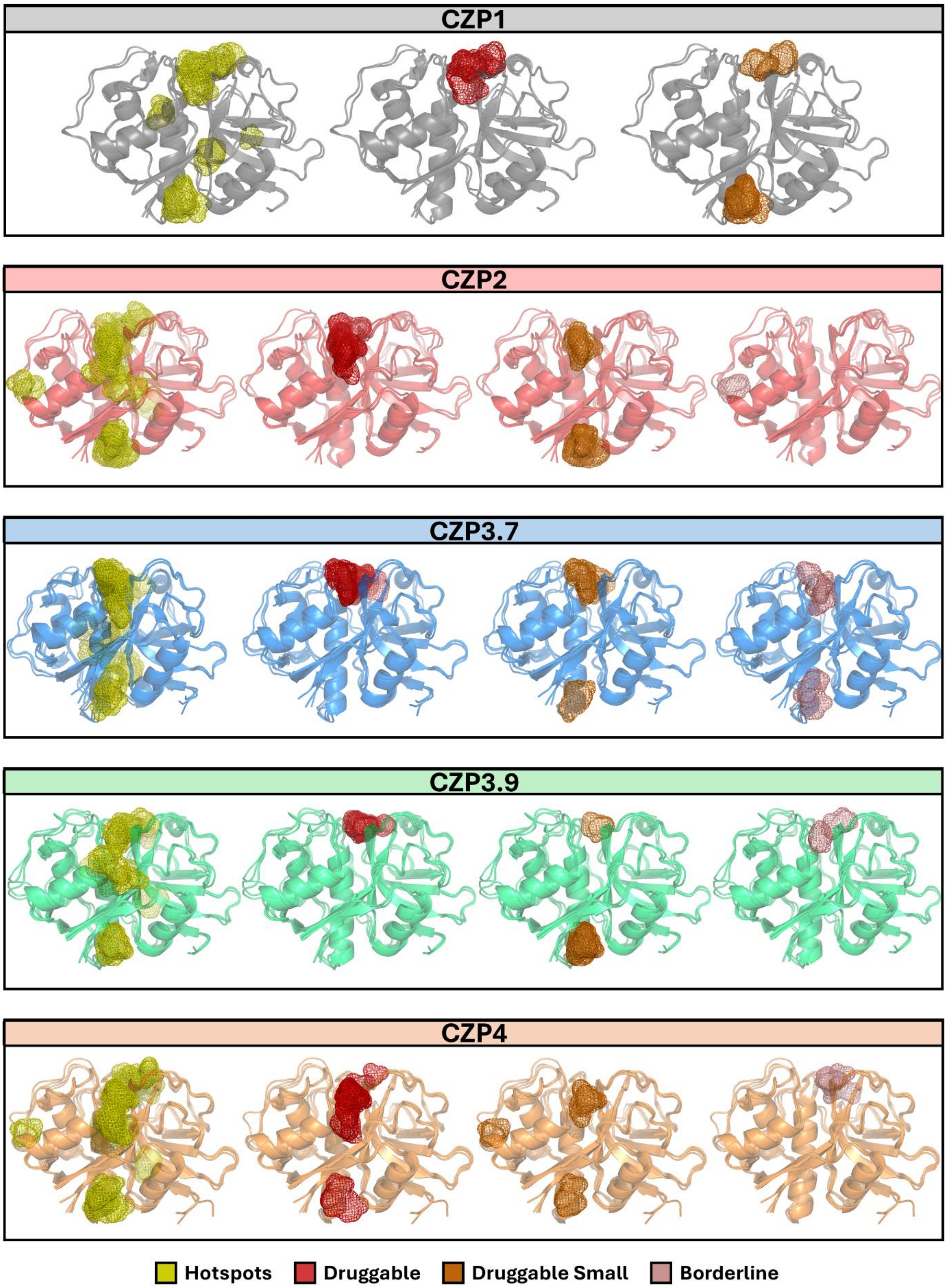

Figure S5. Druggability analysis with the virtual probes from FTMap using DrugPy. DrugPy is a Pymol plugin that finds hotspots (yellow surfaces) in the protein surface from FTMap outcomes and classifies them according to their druggability. Druggability is categorized into three classifications: Druggable (if they exhibit a propensity to bind drug-like

compounds with nanomolar affinity), Druggable low (druggable solely by peptides, macrocycles, or charged compounds), and Borderline (if they demonstrate a tendency to bind drug-like molecules with millimolar or micromolar affinity).

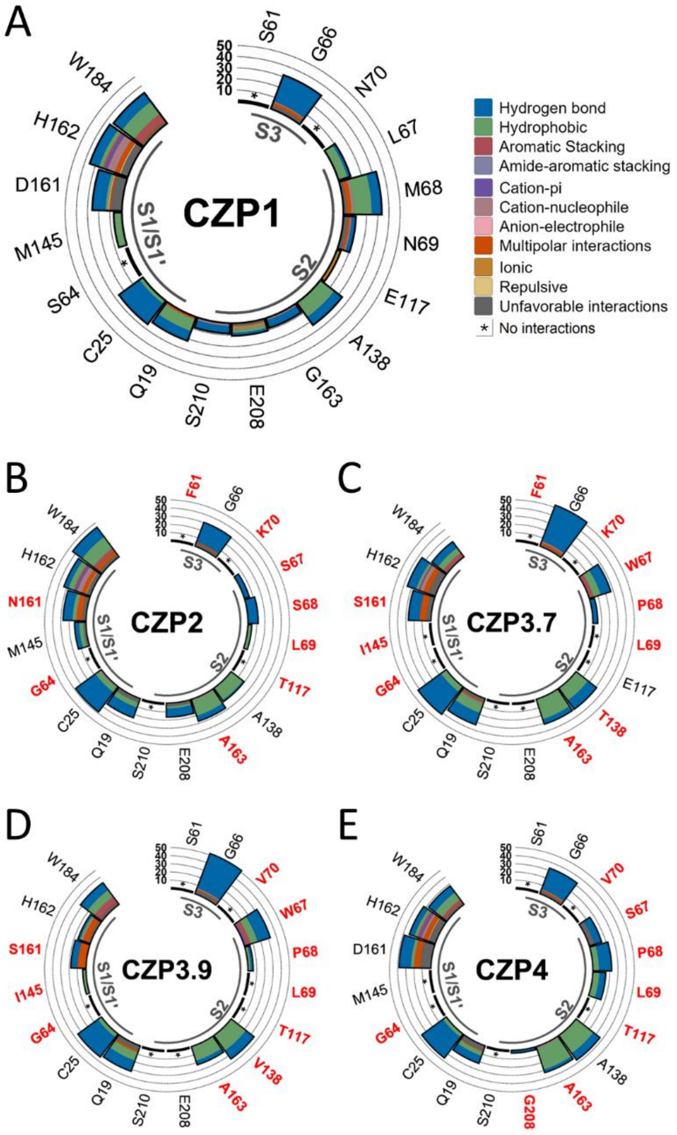

Figure S6. Profile of intermolecular interactions between the virtual probes and active site residues of the PDB ID 1ME3*[1]* for CZP1 and the modeled structures of CZP2, CZP3.7, CZP3.9, and CZP4 from Santos et al.*[2]* Substitutions in cruzipain sequences impact the interactions between the enzyme and the probes. We analyzed the residues from the subsites S3, S2, S1, and S1' for CZP1 (A), CZP2 (B), CZP3.7 (C), CZP3.9 (D), and CZP4 (E). The colors of the bars correspond to the sort of interaction, as stated in (A), and their height is proportional to the interaction frequency. The residues in red are different from the CZP1 residue at the same position.

References

(1) Huang, L.; Brinen, L. S.; Ellman, J. A. Crystal Structures of Reversible Ketone-Based Inhibitors of the Cysteine Protease Cruzain. *Bioorganic & Medicinal Chemistry* **2003**, *11* (1), 21–29. https://doi.org/10.1016/S0968-0896(02)00427-3.

(2) Santos, V. C.; Oliveira, A. E. R.; Campos, A. C. B.; Reis-Cunha, J. L.; Bartholomeu, D. C.; Teixeira, S. M. R.; Lima, A. P. C. A.; Ferreira, R. S. The Gene Repertoire of the Main Cysteine Protease of Trypanosoma Cruzi, Cruzipain, Reveals Four Sub-Types with Distinct Active Sites. *Scientific Reports* **2021**, *11* (1), 18231. https://doi.org/10.1038/s41598-021-97490-2.
